# Cross-Trait Meta-Analysis Reveals a Genetic Link between Inflammation and Aging in Giant Cell Arteritis

**DOI:** 10.14336/AD.2025.0609

**Published:** 2026-08-05

**Authors:** Laura Martínez-Gutiérrez, Inmaculada Rodriguez-Martin, Gonzalo Borrego-Yaniz, Martin Kerick, International GCA Consortium, UK GCA Consortium, Carlo Salvarani, José Hernández-Rodríguez, María Cinta Cid, Miguel Ángel González-Gay, Ann W Morgan, Javier Martín, Lourdes Ortiz-Fernández, Ana Márquez

**Affiliations:** ^1^Institute of Parasitology and Biomedicine López-Neyra, CSIC, Granada, Spain; ^2^Azienda USLIRCCS di Reggio Emilia and Università di Modena e Reggio Emilia, Reggio Emilia, Italy; ^3^Vasculitis Research Unit, Department of Autoimmune Diseases, Hospital Clinic of Barcelona, University of Barcelona, Institut d’Investigacions Biomèdiques August Pi i Sunyer (IDIBAPS), Barcelona, Spain; ^4^Division of Rheumatology, IIS-Fundación Jiménez Díaz, Madrid, Spain; ^5^Department of Medicine, University of Cantabria, Santander, Spain; ^6^Leeds Institute of Cardiovascular and Metabolic Medicine, University of Leeds, Leeds, United Kingdom; ^7^NIHR Leeds Biomedical Research Centre, Leeds Teaching Hospitals NHS Trust, Leeds, United Kingdom

**Keywords:** giant cell arteritis, age-related disease, genetics, senescence, biological aging, cross-trait meta-analysis

## Abstract

Giant cell arteritis (GCA) is a complex inflammatory disease affecting individuals over 50 suggesting a strong link with aging-related immune and vascular changes. However, the precise mechanisms underlying this age-related susceptibility remain poorly understood. Considering the relevance of aging in GCA, genetic factors influencing biological aging markers, such as telomere shortening and epigenetic age acceleration (EAA), might also contribute to its development. This study investigated the shared genetic basis between GCA and these markers to enhance understanding of the role of aging in this vasculitis. Data from approximately 6.6 million variants obtained from previously published genome-wide association studies (GWASs) of GCA (3,498 cases and 15,550 controls), telomere length (472,174 individuals), and EAA (34,710 individuals) were meta-analysed using ASSET. Significant variants (p<5×10^-8^) were functionally annotated, and causal genes were prioritized using FUMA. Potential therapeutic candidates were identified through drug repurposing. This study identified 21 genetic variants shared between GCA and at least one aging marker. Two pleiotropic signals were annotated at *PTPN22* and *PLG*, known risk factors for GCA, whereas the remainder represent potentially new susceptibility loci for this vasculitis. Several prioritized causal genes, such as *SERPING1, SAR1B, SESN1,* and *SMC4*, are involved in both inflammation and senescence, shedding light on the molecular pathways linking aging and GCA. Interestingly, expression levels of some of the prioritized genes *PDE1B*, *ATXN2*, and *CNEP1R1*, were dysregulated in immune cells from active patients. Drug repurposing analysis highlighted promising therapeutic candidates for GCA, including sulfasalazine, an anti-inflammatory agent, and investigational drugs targeting inflammatory pathways like NF-κB. These findings uncover significant genetic overlap between GCA and aging markers, offering insights into shared molecular pathways and potential new therapies targeting both inflammation and cellular senescence.

## INTRODUCTION

Giant cell arteritis (GCA) is the most common immune-mediated vasculitis among adults, and can lead to serious complications such as stroke and permanent vision loss [1]. This multifactorial condition arises from a complex interplay of genetic and environmental factors that trigger an abnormal immune response affecting large and medium-sized blood vessels [2]. In this context, several genetic loci involved in angiogenesis and immune response have been associated with GCA in the largest genome-wide association study (GWAS) conducted on this vasculitis [3].

Although the exact cause of GCA remains unknown, chronological age is the most significant risk factor for its development. Epidemiological studies consistently show that GCA almost exclusively affects individuals over 50 years of age, with incidence peaking between the seventh and eighth decades of life [4]. This age-dependent pattern is likely related to changes resulting from the remodelling of blood vessel walls over time and the aging of the immune system [5]. Specifically, research has revealed an accumulation of cells exhibiting a senescence-associated secretory phenotype (SASP) within the arteries of GCA patients [6,7]. SASP is characterized by the production of pro-inflammatory cytokines and matrix metallo-proteinases, notably interleukin (IL)-6 and matrix metalloproteinase (MMP)-9, which are crucial drivers of GCA pathogenesis by promoting vascular inflammation and tissue remodelling. Additionally, an expansion in an age-related population of circulating CD4+ T-cells displaying a cytotoxic phenotype has been observed in active patients [8]. However, the precise molecular mechanisms linking GCA to the aging process remain to be fully elucidated.

The pace of aging varies among individuals of the same chronological age, likely due to variations in their biological aging processes [9]. Several indicators of biological aging, such as leukocyte telomere length (LTL) attrition and epigenetic age acceleration (EAA), have been found to accurately predict health outcomes and susceptibility to age-related diseases [10,11]. Both LTL shortening and EAA are complex hereditary traits influenced by genetic and environmental factors. Consequently, in recent years, a large number of susceptibility variants have been associated with these aging markers [12,13].

Given the pivotal role that aging plays in both immunosenescence and vascular remodelling, it is plausible that genetic variants influencing biological aging could simultaneously contribute to GCA pathogenesis. The aim of this study was to explore the genetic intersection between this vasculitis and aging by performing a cross-trait meta-analysis of genomic data from GCA and both LTL and EAA markers.

## MATERIALS AND METHODS

### GWAS summary statistics

Summary statistics were retrieved from previously published GWASs conducted for GCA, LTL and EAA [3,12,13] .

The GCA dataset originated from the largest GWAS published to date on this vasculitis, which included data from 10 independent cohorts of European ancestry, encompassing 3,498 cases and 15,550 controls [3]. All GCA patients met the 1990 American College of Rheumatology classification criteria, and the diagnosis was confirmed by positive temporal artery biopsy, positive temporal artery Doppler ultrasonography, or imaging techniques. All patients and controls provided written informed consent in accordance with the tenets of the Declaration of Helsinki.

For LTL, publicly available summary statistics from a large GWAS involving 472,174 participants of European ancestry from the UK Biobank were included in the analysis [12] . With respect to EAA, publicly available summary-level data were obtained from a recent meta-analysis of GWASs on biological aging, which included 34,710 individuals from 28 cohorts of European ancestry [13]. Specifically, we used summary statistics from GWASs that analysed EAA as measured by four different epigenetic clocks: HannumAge acceleration (HannumAA), intrinsic epigenetic age acceleration (IEAA), PhenoAge acceleration (PhenoAA) and GrimAge acceleration (GrimAA). The use of multiple epigenetic clocks allows for a more comprehensive evaluation of biological aging, as each clock captures distinct aspects of the aging process, such as intrinsic cellular aging, phenotypic aging, and mortality risk. Details of each included dataset are provided in Supplementary Table 1.

### Evaluation of local genetic correlation and cross-trait enrichment

We used Local Analysis of [co]Variant Association (LAVA) to assess local genetic correlations between GCA and aging markers across 2,495 independent genomic loci, as previously defined [14].

First, a univariate analysis was performed to identify loci with significant local heritability signals for GCA and at least one aging-related marker, LTL, IEAA, HannumAA, PhenoAA, and/or GrimAA. Only loci reaching significance after Bonferroni correction (p-value<2×10^-5^) were retained for bivariate testing. After excluding the extended major histocompatibility complex (MHC) region (chr6:20-40M), this filtering step resulted in 724 bivariate tests across 460 loci. After bivariate analysis, genomic loci with false discovery rate (FDR)-adjusted p-values < 0.05 were considered to show significant local genetic correlation.

In addition, to depict cross-trait enrichment, we performed conditional quantile-quantile (Q-Q) plots to display the p-value distribution for GCA considering all SNPs, as well as the p-value distribution considering only those SNPs associated with each aging marker (LTL, HannumAA, IEAA, PhenoAA, or GrimAA) at different significance thresholds. SNPs in the extended MHC region were excluded.

### Cross-trait meta-analysis

To identify SNPs showing pleiotropic associations with both GCA and at least one biological aging marker, a subset-based meta-analysis was conducted using the ASSET R package (www.bioconductor.org/packages/release/bioc/html/ASSET.html) [15]. ASSET integrates effect sizes and standard errors from GWAS summary statistics to systematically evaluate the associations of genetic variants across all possible subsets of traits. This method identifies the subset that contributes most significantly to the overall association signal and quantifies its statistical significance. For signals displaying opposite effects on different traits, ASSET allows for a separate subset search for positively and negatively associated traits, thereby providing significance values for each subset direction.

Given the sample overlap among the different EAA datasets, we conducted four independent cross-trait meta-analyses, pairing GCA and LTL with each of the EAA measures. This approach aimed at reducing the risk of overestimating correlations in association tests, which could result in false positives.

The HLA region (Chr6: 20-40 MB) was excluded from the analysis because of its complexity and well-established associations with GCA. Furthermore, we restricted our analysis to common variants with a minor allele frequency (MAF) above 1%. Following cross-trait meta-analysis, SNPs with a p-value<5x10^-8^ in the overall association were considered as statistically significant. For SNPs exhibiting opposite effects on different traits, only those with p-values<0.05 in both the positively and negatively associated subsets were considered significant.

Among the significant SNPs, we focused on the signals where both GCA and at least one aging marker contributed to the association. Given the substantial sample size of the GWAS for the aging markers compared with the smaller sample size for GCA, a p-value<0.01 in the GCA GWAS was required to ensure the identification of relevant signals for this vasculitis. When multiple SNPs were statistically significant within the same locus, the lead SNPs were selected as those that included the largest number of traits in the best subset. Additionally, we considered only SNPs that were included in at least one of the largest GCA cohorts (Spain and/or UK) to ensure robust representation. Finally, genome-wide associated SNPs within the same pleiotropic locus were considered independent if they displayed low linkage disequilibrium (LD) (r² < 0.1 and D' < 0.5) within a 1000-kb interval.

### Polygenic risk score for GCA prediction

We evaluated whether the significant SNPs identified in this study could enhance the performance of a previously developed polygenic risk score (PRS) for predicting GCA genetic predisposition [3]. In addition to the 11 variants included in the original PRS, we incorporated into the model all newly identified, significant, and independent lead SNPs. Before inclusion, we verified that these variants were not in high LD with any of the SNPs in the original model, to avoid redundancy and potential model inflation.

Once the revised PRS was constructed, we applied it to the same subset of the cohort (1,034 cases and 1,294 controls) as in the experimental design of the original GWAS, to enable direct comparison of the prediction results between studies. To assess the overall predictive ability of the model, we calculated the area under the receiver operating characteristic curve (AUC). Additionally, we evaluated the model’s capacity to identify individuals at particularly high genetic risk by analyzing the proportion of cases within the top 10% of the PRS distribution.

### Functional annotation of genetic variants and gene prioritization

Functional Mapping and Annotation (FUMA) of GWAS (https://fuma.ctglab.nl/) was employed to annotate and prioritize likely causal genes within each pleiotropic locus identified in the cross-trait meta-analysis [16]. Specifically, the SNP2GENE function in FUMA maps independent significant SNPs and their proxies (r^2^> 0.9) to potential causal genes on the basis of several criteria, including the physical proximity between the SNP and the potential gene and the effects of the SNP on gene expression in GCA-relevant tissues (such as whole blood, immune cells, and vascular tissue). To further refine the SNP-to-gene mapping, protein quantitative trait locus (pQTL) data, splicing quantitative trait locus (sQTL) data and V2G scores from the Open Target Genetics Platform were incorporated.

### Differential expressions of prioritized genes in monocytes and CD4+ T cells

Genes prioritized by FUMA from the shared genetic signals across GCA, LTL, and EAA were evaluated in previously published transcriptomic data [17,18], which included gene expression profiles of peripheral blood CD4^+^ T cells and monocytes from patients with active GCA, patients in remission without treatment, patients in remission with treatment, and healthy controls. To assess whether prioritized genes were differentially expressed, we queried the differential expression results from the original analysis. Genes were considered differentially expressed if they showed an FDR < 0.05. Comparisons involving patients in remission with treatment were excluded to avoid confounding effects of therapy.

### Regulatory elements enrichment analysis

We explored the tissue-specific enrichment of pleiotropic variants across different regulatory elements by applying GoShifter (https://github.com/immunogenomics/goshifter) [19]. Specifically, we focused on nine histone marks associated with active promoters (H3K4me2, H3K4me3, H3K9ac, H3K4ac), enhancers (H2BK20ac, H3K27ac, H3K4me1), and actively expressed genes (H3K79me1, H2BK15ac) from the Roadmap Epigenomics project. The analysis was performed using several approaches. First, we considered all suggestive pleiotropic variants collectively. Then, we conducted separate analyses for LTL and EAA, distinguishing whether the effect of the variants was in the same direction for both the trait and GCA or if they exhibited opposing effects. Only functional annotations from vascular and immune tissues or cell types were included.

### Gene set enrichment analysis of aging-related genes

EnrichR [20] was used to assess whether the set of prioritized genes was significantly enriched for aging-related genes based on three gene-set libraries comprising genes differentially expressed during aging: GTEx Aging Signatures 2021, Aging_Perturbations_from_GEO_ down, and Aging_Perturbations_from_GEO_up. Terms with a p-value < 0.05 and with a minimum overlap of three genes were considered statistically significant.

### Drug repurposing analysis

To explore potential new treatment options for GCA, we conducted a drug repurposing analysis using genes prioritized as potentially causal after functional annotation. We then examined the DrugBank database [21] for available drugs targeting any of these genes. In order to propose viable treatments for GCA, we focused on the mechanism of action of the drugs listed in DrugBank and supplemented this information with a manual review of the relevant literature.

**Table 1:** Independent genetic variants that reached genome-wide significance after cross-disease meta-analysis and met the established association criteria.

Region	bp	SNP	Closest gene	EA	P-value*	OR (95% CI)	Best subset
1p13.2	113849937	rs1217404	*PTPN22*	T	3.97E-14	1.08 (1.02-1.15) | 0.99 (0.98-0.99)	GCA | LTL EAA
2p14	65413095	rs10190233	*SPRED2*	A	1.29E-08	1.01 (1.01-1.02) | 0.92 (0.86-0.98)	LTL EAA | GCA
3q25.33	160352276	rs12630770	*IFT80*	A	4.23E-16	1.17 (1.09-1.25) | 0.98 (0.98-0.99)	EAA | GCA LTL
4p16.3	2195586	rs7666644	*POLN*	T	1.78E-14	1.16 (1.04-1.30) | 0.98 (0.97-0.98)	GCA | LTL EAA
4q24	102528926	rs230533	*NFKB1*	A	7.03E-10	1.16 (1.09-1.23) | 0.87 (0.81-0.92)	GCA | EAA
5q31.1	134502489	rs1981627	*JADE2*	A	5.39E-09	1.15 (1.08-1.22) | 0.99 (0.99-1.00)	EAA | GCA LTL
5q31.1	135371383	rs1393082	*MACROH2A1*	A	2.47E-09	1.16 (1.10-1.22) | 0.99 (0.99-1.00)	GCA EAA | LTL
6q21	109330017	rs12200757	*CD164*	T	1.26E-10	1.01 (1.01-1.02) | 0.89 (0.83-0.95)	GCA LTL | EAA
6q26	160671406	rs11751347	*LPA*	T	2.43E-08	1.13 (1.02-1.25) | 0.74 (0.66-0.82)	GCA | EAA
7q32.1	129004479	rs3807304	*TNPO3*	T	1.16E-09	1.02 (1.01-1.02) | 0.88 (0.82-0.96)	LTL EAA | GCA
8q12.1	56314526	rs4737427	*SDR16C5*	A	4.41E-09	1.02 (1.01-1.02) | 0.87 (0.79-0.96)	LTL EAA | GCA
11q12.1	57360662	rs11229011	*P2RX3*	A	1.28E-08	1.18 (1.12-1.25)	GCA EAA
12q13.13	54342686	rs4326844	*COPZ1*	A	1.08E-08	1.11 (1.05-1.17) | 0.99 (0.99-0.99)	GCA EAA | LTL
12q24.12	111466567	rs4766578	*ATXN2*	A	3.92E-17	1.02 (1.01-1.02) | 0.91 (0.84-0.98)	GCA LTL | EAA
16p13.3	3703680	rs17256002	*TRAP1*	A	9.98E-09	1.18 (1.05-1.31) | 0.98 (0.97-0.99)	GCA EAA | LTL
16q12.1	50120902	rs6500291	*HEATR3*	C	2.68E-11	1.01 (1.01-1.02) | 0.91 (0.85-0.96)	LTL | GCA EAA
16q23.1	74477970	rs147022170	*GLG1*	C	2.82E-14	1.03 (1.02-1.04) | 0.83 (0.74-0.95)	LTL | GCA
17p13.3	2235364	rs12450118	*SMG6*	C	4.03E-09	1.12 (1.06-1.18) | 0.99 (0.99-0.99)	GCA | LTL EAA
18p13.32	657352	rs2853741	*TYMS*	T	5.00E-10	0.99 (0.98-0.99)	GCA LTL EAA
18q12.3	44541810	rs117598048	*SETBP1*	G	3.41E-17	1.04 (1.03-1.05) | 0.50 (0.38-0.65)	GCA LTL | EAA
18q21.2	54354217	rs5825069	*STARD6*	A	3.34E-17	1.02 (1.01-1.02)	GCA LTL

Traits that contribute most to the association signal define the best subset. Identified associations among these traits that have not been previously reported are shown in bold. SNP, single-nucleotide polymorphism; bp, base pair; OR, odds ratio; EA, effect allele; CI, confidence interval; GCA, giant cell arteritis; LTL, leukocyte telomere length; EAA, epigenetic age acceleration.

* For SNPs that were significant in more than one of the cross-trait meta-analyses, the most significant p-value across analyses is reported. The p-values and ORs from each individual cross-trait meta-analysis are available in https://doi.org/10.5281/zenodo.16743049.

**Figure 1.**
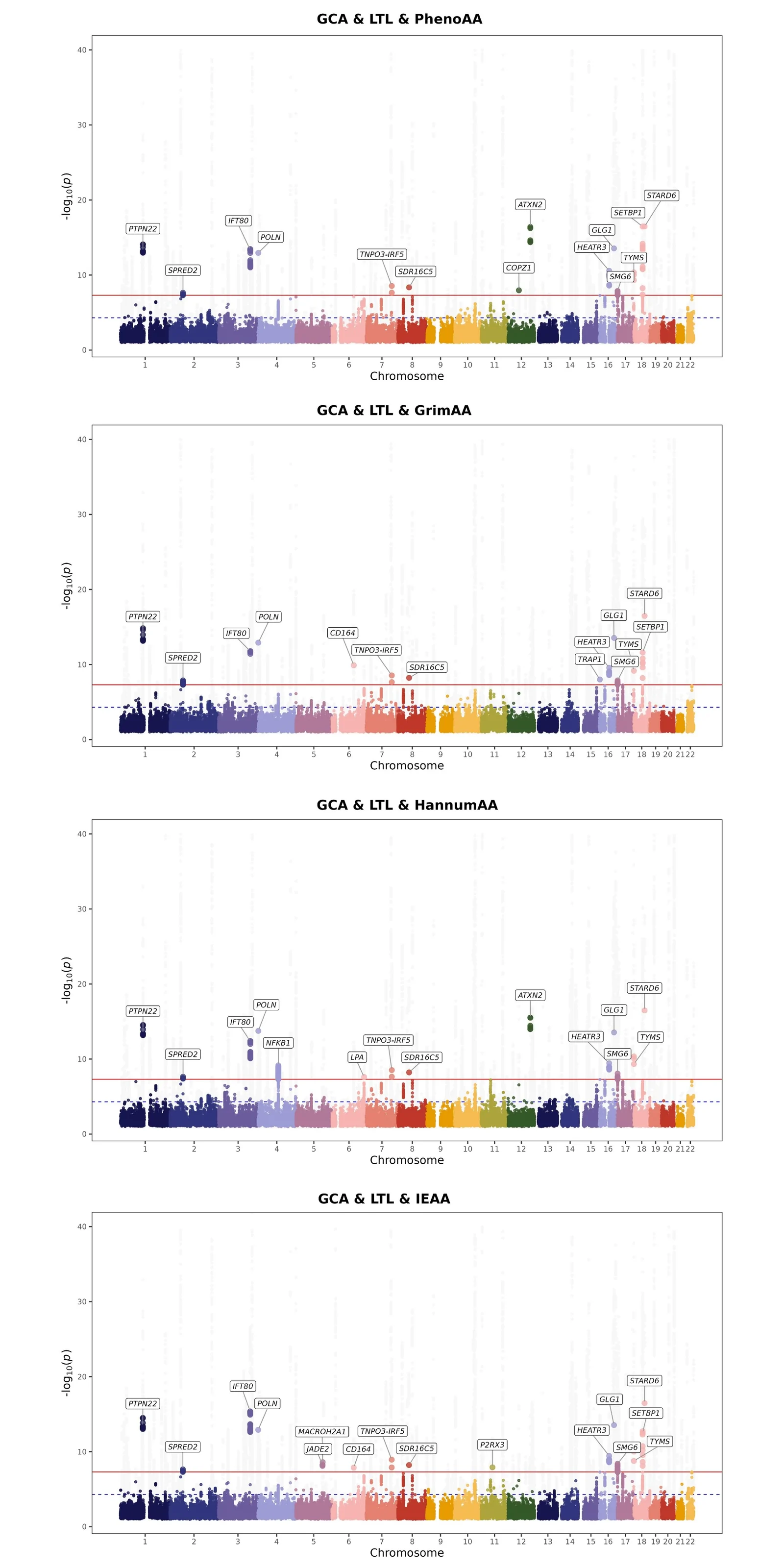
**Manhattan plots of the cross-trait meta-analyses for giant cell arteritis (GCA), leukocyte telomere length (LTL), and the estimated epigenetic age acceleration according to GrimAge acceleration (GrimAA), PhenoAge acceleration (PhenoAA), HannumAge acceleration (Hannum AA), and intrinsic epigenetic age acceleration (IEAA)**. The x-axis represents the genomic position by chromosome, whereas the y-axis represents the negative logarithmic transformation of the p-value. Each point corresponds to an analysed SNP. The red line indicates the significance threshold at the genome-wide level (p-value < 5x10^-8^). For each of these highlighted signals, the closest gene is indicated.

## RESULTS

### Cross-trait enrichment between GCA and biological aging markers

The conditional Q-Q plots revealed an enrichment of SNPs associated with GCA when conditioned on the significance of the different aging markers (LTL, HannumAA, IEAA, PhenoAA, and GrimAA) (Supplementary Fig. 1). In all cases, we observed a deviation from the expected distribution under the null hypothesis, which supports the presence of shared genetic architecture between GCA and aging markers. Specifically, the enrichment effect appeared more pronounced, particularly at more stringent significant thresholds, when conditioning on LTL and HannumAA significance, suggesting a stronger polygenic overlap between GCA and these aging markers.

Additionally, we identified three loci showing FDR-significant local genetic correlations between GCA and aging markers (Supplementary Fig. 2 and Supplementary Table 2). Furthermore, 105 additional loci showed nominal evidence of local genetic correlation across 94 genomic regions (Supplementary Fig. 2), further supporting the presence of a shared genetic component between GCA and biological aging markers.

### Cross-trait meta-analysis

Four cross-trait meta-analyses, including a total of 6,641,839 SNPs, were conducted using summary statistics from GWASs of GCA, LTL, and each EAA estimate (HannumAA, IEAA, PhenoAA, and GrimAA) separately. After excluding the HLA region, 280 genetic variants distributed across 20 genetic regions were associated with GCA and at least one biological aging marker (Fig. 1). According to the LD pattern, 21 genetic variants located within these specific loci were found to be independently associated (Table 1).

**Figure 2.**
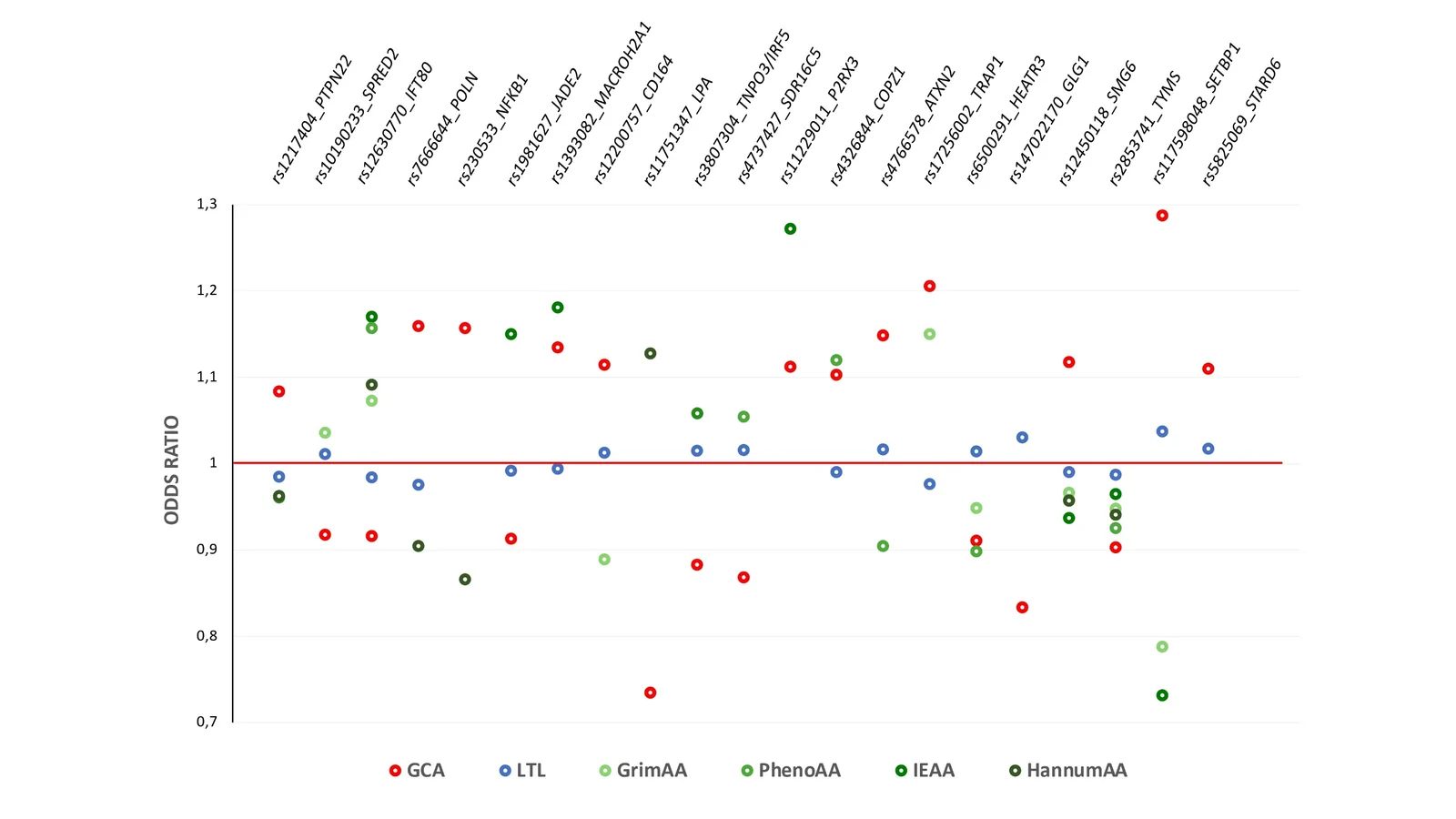
**Effects of the pleiotropic variants on each of the analysed traits, giant cell arteritis (GCA), leukocyte telomere length (LTL), and markers of accelerated epigenetic aging (GrimAge acceleration (GrimAA), PhenoAge acceleration (PhenoAA), HannumAge acceleration (HannumAA), and intrinsic epigenetic age acceleration (IEAA), represented in different colours**. The ORs of the variants for the traits included in the best subset are shown. The red line is positioned at OR = 1, which corresponds to an effect size where the variant has no effect on the trait.

Some pleiotropic loci appeared to be associated with only one specific clock, while others were shared across multiple clocks (Supplementary Table 3); importantly, the effects of the shared variants among clocks consistently showed the same direction across them (Fig. 2). Considering this, to simplify our results, we refer to EAA as the association with any of the clocks rather than specifying individual associations.

**Figure 3.**
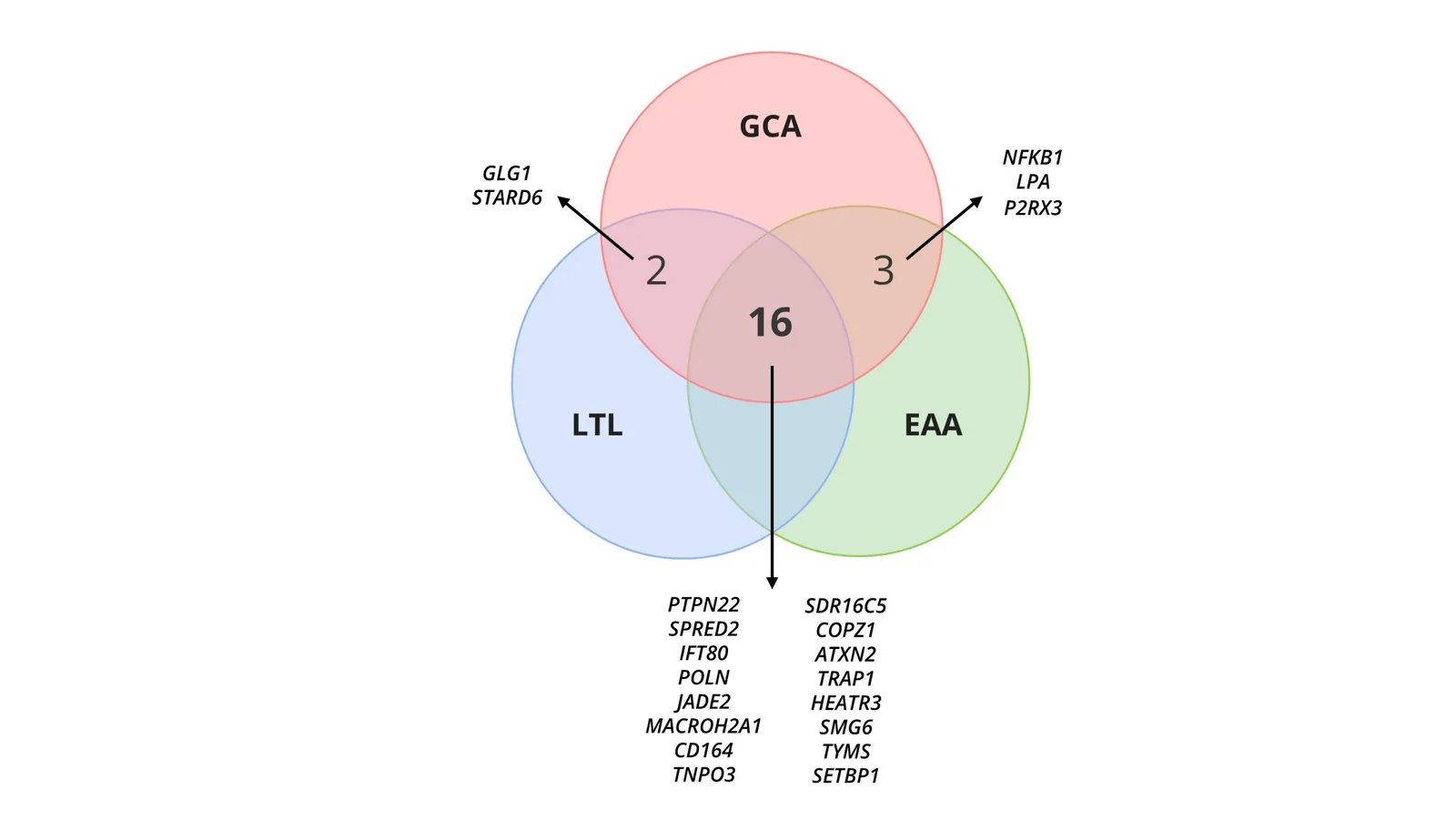
Venn diagram showing the overlap of shared signals across the analysed traits, giant cell arteritis (GCA), leukocyte telomere length (LTL), and epigenetic age acceleration (EAA), after cross-trait meta-analysis, along with the nearest genes to the shared variants.

As shown in Figure 3, most of the pleiotropic signals, specifically 16 out of 21, were shared among the three traits. Notably, two of the shared signals corresponded to loci previously associated with GCA, *PTPN22* and *PLG*, whereas the other 19 loci potentially represent new susceptibility factors for this vasculitis. Additionally, five of these pleiotropic signals, located at the genomic regions 2p14, 5q31.1, 12q13.13, and 17p13.3, have not been previously described for any of the analysed traits and may represent new candidate susceptibility loci for both GCA and the two aging markers (Table 1 and Supplementary Table 3). Notably, 12 out of the 21 signals (57%) had similar effects in GCA and at least one of the aging markers, whereas 9 genetic variants had opposite effects on GCA compared with LTL and/or EAA (Table 1 and Supplementary Table 3).

### Improved ability for GCA genetic prediction

Of the 21 significant lead SNPs identified in this study, 20 were incorporated into a revised PRS model for GCA prediction. A detailed list of these SNPs, including effect alleles and beta coefficients, is provided in Supplementary Table 4. The only exception was rs11751347, which had already been included in the original PRS. The revised PRS, comprising a total of 31 SNPs, was evaluated in a subset of the cohort; however, only 23 of these variants could be used for assessing prediction due to limitations in the genotypic data of this cohort.

The revised PRS demonstrated a modest improvement in overall predictive performance, leading to an AUC of 0.624 compared to 0.617 in the original model. Similarly, the model's ability to identify individuals at high genetic risk for GCA was slightly enhanced. Individuals in the top 10% of the PRS distribution showed an increased risk of developing GCA (OR = 2.93 [95% CI: 2.20-3.91]), compared to an OR of 2.87 [95% CI: 2.15-3.82] observed in the original model. These findings suggest that incorporating SNPs shared between GCA and aging can improve the predictive accuracy of PRS models for GCA genetic susceptibility.

### Functional effects of pleiotropic variants and gene prioritization

The functional annotation of the lead SNPs and their proxies via FUMA and Open Targets Genetics showed that none of the lead SNPs were located in coding regions. However, three of these SNPs, at the 4p16.3, 12q24.12, and 17p13.3 loci, were strongly linked (r^2^≥0.9) to exonic variants (Supplementary Fig. 3), including three missense (rs9328764, rs10011549, and rs10018786) and three synonymous (rs6830513, rs2022302, and rs10002583) variants at *POLN*, a missense variant (rs3184504) at *SH2B3*, and a missense variant (rs1885986) at *SMG6*. This last SNP showed a deleterious effect according to SIFT (0.03) and PolyPhen (0.987). These results suggest that these genes are the strongest candidates for being causal within these loci.

Notably, nearly all the pleiotropic SNPs and/or their proxies, with only one exception, appeared to exert regulatory effects on gene expression in immune cells, whole blood, and/or arterial tissue (Supplementary Fig. 3 and Supplementary Table 5 (see https://zenodo.org/records/16743049), as well as on splicing (Supplementary Fig. 3 and Supplementary Table 6). Additionally, genetic variants at four of these shared signals act as pQTLs influencing the levels of proteins encoded by *MANBA*, *PLG*, *PENK*, and *SERPING1*, suggesting their potential functional relevance (Supplementary Fig. 3 and Supplementary Table 7).

**Figure 4.**
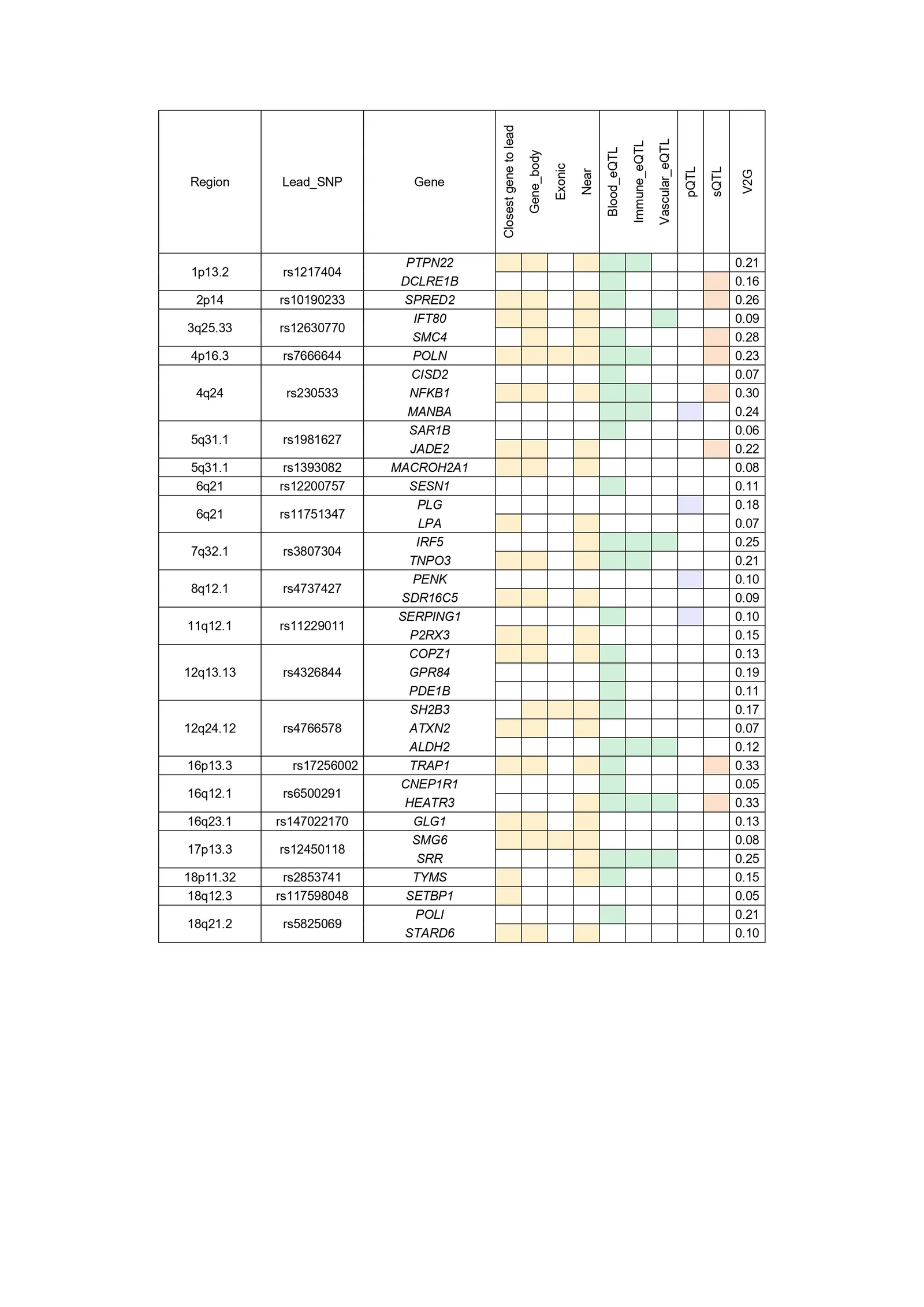
**Functional annotation and gene prioritization for the identified pleiotropic signals**. For each locus, only the top prioritized genes are displayed. The colours indicate the SNPs that overlap with the considered functional annotations for each specific gene. Different colours indicate different categories of annotations, including the genomic location of the genetic variants, their correlation with gene expression, splicing changes, and protein levels, and their V2G score from Open Target Genetics. SNP, single-nucleotide polymorphism; eQTL, expression quantitative trait loci; sQTL, splicing quantitative trait loci; pQTL, protein quantitative trait loci; V2G, Variant-to-Gene.

On the basis of the annotation results, we used FUMA and additional data from Open Targets to propose the most likely causal genes of these pleiotropic signals, prioritizing 94 genes as potential causal candidates for further investigation (Supplementary Fig. 3). A subset of these prioritized genes, together with the key functional annotations of the corresponding variants, are summarized in Figure 4.

### Differential expression of prioritized genes

Three of the genes prioritized using FUMA were found to be differentially expressed in immune cells from patients with GCA. Specifically, *PDE1B* and *CNEP1R1* were significantly upregulated in monocytes from patients with active GCA compared to patients in remission (P_FDR_= 0.04, logFC=0.58; P_FDR_=0.009, logFC=0.43, respectively), while *ATXN2* was significantly downregulated in CD4+ T cells from active patients compared to healthy controls and patients in remission (P_FDR_=0.03, logFC=-0.32).

### Enrichment in cell-type-specific histone marks

GoShifter was used to identify trait-relevant tissues and cell types. We explored enrichment patterns at tissue-specific histone marks for all suggestive pleiotropic variants, as well as those exhibiting the same or opposite effect between GCA and at least one of the aging markers individually.

**Figure 5.**
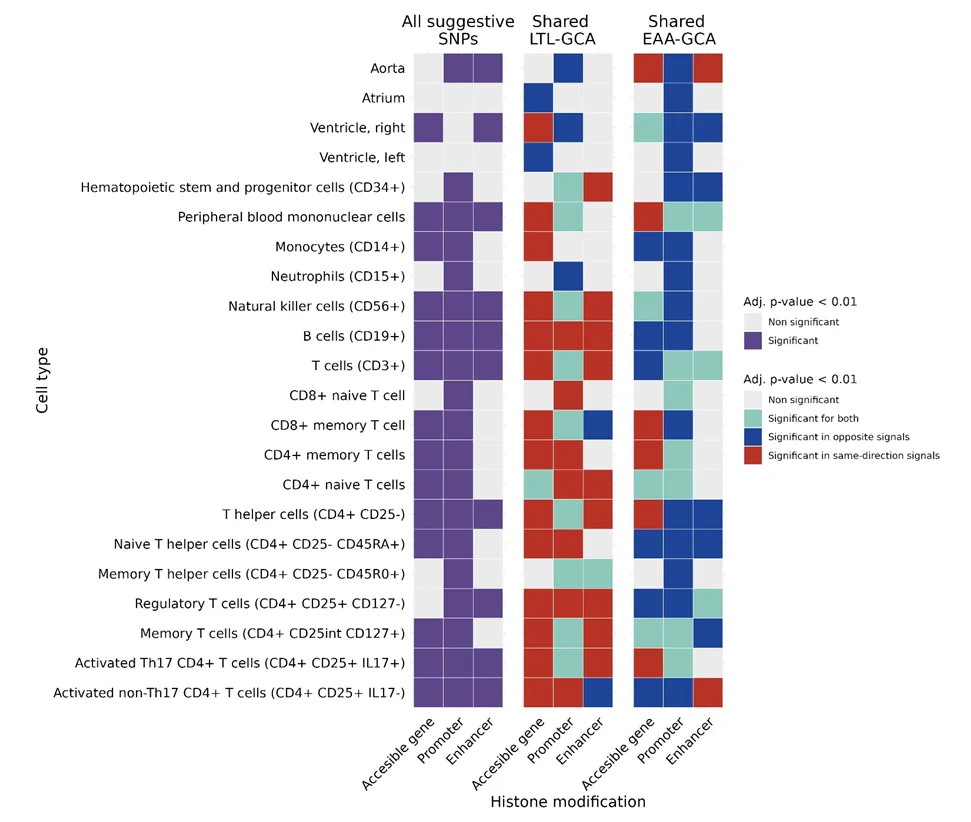
**Results of the histone mark enrichment analysis for three sets of genetic variants: all suggestive pleiotropic variants (left), variants shared between giant cell arteritis (GCA) and leukocyte telomere length (LTL) (middle), and variants shared between GCA and epigenetic age acceleration (EAA) (right)**. The y-axis lists the analysed cell types, while the x-axis represents histone modifications associated with accessible genes, promoters, and enhancers. Colours indicate the statistical significance of the enrichment (adjusted p-value < 0.01) and whether the enrichment signal in each specific cell type was observed for concordant effects between GCA and LTL or EAA, for discordant effects, or for both.

A strong enrichment in various histone marks was evident across nearly all explored immune cell types, as well as in the aorta and right ventricle, when considering all pleiotropic SNPs with suggestive associations in the cross-trait meta-analysis (Fig. 5 and Supplementary Fig. 4). However, when shared SNPs with concordant or discordant effects in GCA and LTL were analysed separately, different enrichment patterns emerged. While concordant signals were enriched across nearly all immune cell types but not in vascular tissues (Fig. 5 and Supplementary Fig. 5), and spanned multiple histone marks, opposite signals showed enrichment in all analysed vascular tissues (aorta, ventricle, and atrium) but only in a few immune cell types, particularly in neutrophils, and primarily for the H3K4ac histone mark (Figure 5 and Supplementary Fig. 6). In contrast, the opposite trend was observed for SNPs shared between EAA and GCA, where discordant signals showed stronger enrichment in immune cell types than concordant signals (Fig. 5, Supplementary Fig. 7 and 8).

### Enrichment in aging-related genes

To explore the relationship between our prioritized genes and aging, we evaluated three age-related gene-set libraries using EnrichR. According to the Aging_Perturbations_from_GEO_down and Aging_ Perturbations_from_GEO_up libraries, a significant overlap was observed between our prioritized genes and genes showing age-associated expression changes in CD4+ T lymphocytes (Supplementary Tables 8 and 9). Notably, 46 prioritized genes were dysregulated with age in relevant cell types, including CD4+ T lymphocytes, fibroblasts, dendritic cells, and macrophage progenitors (Supplementary Tables 8 and 9).

Although no significant enrichment was detected in the GTEx Aging Signatures 2021 library, several of our prioritized genes, including *PRG2*, *PLG*, *PRG3*, *SDR16C5*, *NFE2*, and *GTSF1*, were differentially expressed in the blood of older individuals. In addition, according to the GTEx Aging Signatures, *PENK* showed age-related upregulation in blood vessels (Supplementary Table 10).

### Drug repurposing opportunities for GCA treatment

Finally, we performed a drug repurposing analysis to identify existing drugs indicated for other conditions that might be repurposed for the treatment of GCA. Through this approach, we identified 70 drugs potentially suitable for repositioning in GCA, targeting proteins encoded by 15 of the prioritized genes (Supplementary Table 11). Among these drugs, 40 have already been approved for the treatment of other diseases, while the remaining 30 are still in experimental stages.

## DISCUSSION

Our cross-trait meta-analysis provides the first evidence of a genetic overlap between GCA and two biomarkers of biological aging, LTL and EAA, identifying 21 shared loci. Although most of these loci are located in non-coding regions of the genome, functional annotations and histone mark enrichment suggest they exert regulatory effects by modulating gene expression in cell types and tissues relevant to GCA, thereby enabling the prioritization of potential causal genes.

Over half of the pleiotropic variants (57%) showed concordant effects on GCA and at least one aging marker, thus indicating the existence of shared molecular mechanisms between them. Shared SNPs showing concordant effects in GCA and LTL were enriched in histone marks across multiple immune cell types, suggesting that genetic variants conferring risk to longer telomeres may also increase GCA risk by enhancing the survival of inflammatory cells. On the other hand, shared variants with concordant effects in GCA and EAA were enriched primarily in histone marks across different vascular tissues and some immune cell types, suggesting that SNPs associated with increased epigenetic age may contribute to GCA risk by promoting vascular and immune senescence.

Interestingly, expression levels of three prioritized genes within the concordant signals, *PDE1B*, *ATXN2*, and *CNEP1R1*, were dysregulated in immune cells from GCA patients compared with those from controls and/or patients in remission. Notably, these genes are implicated in the regulation of inflammatory processes. *PDE1B*, overexpressed in GCA monocytes, is regulated by granulocyte-macrophage colony-stimulating factor and promotes monocyte-to-macrophage differentiation [22,23]. *ATXN2*, downregulated in CD4+ T cells from GCA patients, acts as a repressor of mTORC1 signalling [24,25], a pathway critical for T-cell polarization and inflammation in GCA. Activation of mTORC1 has been demonstrated in the endothelium of the arterial wall and in Th1/Th17 cells from GCA lesions [5,26,27]. Moreover, mTORC1 has been linked to aging and longevity [28,29]. *CNEP1R1*, also overexpressed in GCA monocytes, forms an active complex with the serine/threonine protein phosphatase CTDNEP1, which dephosphorylates and activates lipin-1 and -2. These enzymes are involved in macrophage-mediated inflammation by regulating lipid metabolism in this cell type [30].

In addition, several prioritized genes were enriched in aging-associated transcriptional signatures of CD4^+^ T cells and other GCA-relevant cell types, reinforcing the connection between age-related immune remodelling and disease risk. Indeed, many of the prioritized genes at these concordant loci are implicated in pathways linking inflammation and aging, making them strong candidates for involvement in the pathogenesis of GCA.*SERPING1* (11q12.1) encodes the C1-inhibitor, a central regulator of the complement cascade, which is also involved in regulating vascular permeability and suppressing inflammation. It is also functionally linked to the GCA-associated gene *PLG*, as it participates in the inactivation of plasmin and tissue plasminogen activator [31]. Interestingly, *SERPING1* expression increases with age and correlates with the levels of IL-6, a key cytokine that drives inflammation in patients with GCA [32,33].

Another key finding involves the 5q31.1 and 6q21 loci, where *SAR1B* and *SESN1*, respectively, were prioritized. Both genes encode leucine sensors involved in mTORC1 signalling [34-37], a relevant pathway in GCA pathogenesis and senescence, as previously mentioned.

Additional prioritized genes, including *SMC4* and *MACROH2A1*, further highlight the interplay between immune regulation and aging processes in GCA. *SMC4* (3q25.33) encodes a condensin that contributes to chromosomal organization and has roles in telomere stability, cellular senescence through the activation of SASP genes, and p53-mediated pathways [38-40]. It also promotes inflammatory responses by enhancing innate immunity through the activation of NF-κB essential modulator (NEMO) [41]. *MACROH2A1* (5q31.1) encodes a histone variant that may influence the epigenetic regulation of genes involved in inflammatory responses, including cytokines, chemokines, and metalloproteases that constitute the SASP [42,43]. Therefore, these genes may contribute to the pathogenesis of GCA by regulating both cellular senescence and inflammation, processes that are tightly interconnected and play pivotal roles in this vasculitis.

Notably, 43% of the identified pleiotropic variants showed opposing effects in GCA relative to aging markers. In this regard, discordant variants between GCA and LTL were enriched in histone marks primarily in vascular tissues and some immune cell types, suggesting that variants associated with shorter telomere length could increase the risk of GCA by promoting vascular and immune senescence. Regarding genetic variants with opposite effects in GCA and EAA, enrichment was observed across nearly all explored tissues and cell types. However, since the biological foundations of epigenetic clocks remain largely unelucidated, it is challenging to explain the divergent effects observed between GCA and EAA.

A possible explanation is that variants with opposite effects may influence different biological processes depending on the context, thus acting in distinct ways in GCA and aging. For example, at the 1p13.2 signal, two relevant genes, *PTPN22* and *DCLRE1B*, were prioritized, showing opposite effects on GCA with respect to LTL and EAA. Although the lead SNP within this locus (or its proxies) acts as an eQTL for both *PTPN22* and *DCLRE1B* in blood, it is specifically an eQTL for *PTPN22* in certain immune cells. In addition, tissue-specific expression data show that *PTPN22* and *DCLRE1B* exhibit markedly different expression patterns: *PTPN22* shows high expression in whole blood and EBV-transformed lymphocytes but is virtually not expressed in arterial tissues, whereas *DCLRE1B* shows the opposite pattern, with higher expression in arteries compared to blood (Supplementary Fig. 9). These findings suggest that *PTPN22*, involved in the immune response and autoimmune diseases [44], could mediate its effects on GCA through immune-related mechanisms, whereas *DCLRE1B*, which plays a role in telomere maintenance [45], may be more relevant to vascular aging.

Similar expression divergence is observed in other loci showing opposite effects between GCA and aging markers (Supplementary Fig. 9), reinforcing the idea that cell type-specific regulatory contexts contribute to the dual roles of these variants. A comparable pattern was observed at other loci containing inflammatory genes, such as 4q24, where several potential causal genes were identified. Interestingly, the signal in this region had opposite effects on GCA and EAA, highlighting its complex involvement in inflammatory and aging-related processes. Prioritized genes at this locus included *NFKB1*, a crucial transcription factor involved in inflammation that plays a central role in the regulation of the SASP [46], *MANBA*, a glycosidase involved in cellular senescence [47,48] and *CISD2*, which is associated with age-related diseases and whose deficiency leads to premature aging [49,50].

A proposed hypothesis for the relationship between advanced age and the development of GCA is arterial wall remodelling associated with aging. Interestingly, one of the shared variants (rs11751347), which exhibited opposite directional effects in GCA and EAA, was annotated to *PLG*, an established susceptibility locus for this vasculitis [3]. *PLG* encodes plasminogen, the precursor to plasmin, which plays a critical role in angiogenesis, a key process in the vascular remodelling observed in GCA [51]. However, the opposing effects observed for this locus in GCA and EAA suggest that the *PLG*-related mechanisms underlying aging and inflammation in GCA may diverge or involve distinct pathways. In this sense, *PLG* has also been linked to cellular senescence through the regulation of plasminogen activator inhibitor 1 (PAI-1), a key mediator of senescence that modulates the extracellular proteolysis of SASP components in various cell types and contributes to vascular aging [52,53]. Additionally, urokinase-type plasminogen activator receptor (uPAR), which activates plasminogen to plasmin, has been identified as a cell surface marker of senescent cells, and its soluble form is secreted as part of the SASP [54].

GWAS findings are increasingly recognized as valuable for uncovering opportunities to repurpose existing drugs [55]. In this context, our drug repurposing analysis identified several potential candidates for the treatment of GCA, including the approved drug sulfasalazine, an anti-inflammatory molecule indicated for managing inflammatory diseases such as rheumatoid arthritis. Although the exact mechanism of action of this drug is not fully understood, it is thought to be mediated through the inhibition of various inflammatory molecules, including NF-κB [56].

Several of the genes prioritized in our study have been proposed as potential drug targets. For instance, strategies aimed at inhibiting components of the plasminogen system have been developed and suggested as a therapeutic approach for age-related diseases. In this context, pharmacological targeting of PAI-1 has demonstrated efficacy in reducing vascular remodelling [57-59], and uPAR has been effectively targeted for the clearance of senescent cells [60,61].

Our findings also suggest a potential role in GCA for genes involved in the mTORC1 pathway. For example, *SESN1* acts as a negative regulator of mTORC1 and contributes to oxidative stress response and cellular senescence [34]. Thus, pharmacological modulation of mTORC1, either directly or indirectly, may influence both immune activation and vascular aging processes relevant to GCA. Notably, mTOR inhibitors have been proved useful in controlling inflammation and are currently being evaluated as therapeutical options for inflammatory immune-mediated diseases, including systemic lupus erythematosus, rheumatoid arthritis, and Takayasu’s arteritis, a large-vessel vasculitis, suggesting their potential use in GCA [62,63]. Nevertheless, targeting senescence pathways such as mTORC1 may have adverse effects, including immune suppression or metabolic dysregulation, underscoring the importance of carefully evaluating the benefit-risk profile of these approaches in GCA.

Despite these insights, this study has some limitations that should be acknowledged. Although cross-trait meta-analysis is a powerful approach to identify shared genetic architecture, it does not provide direct mechanistic evidence for the observed associations. Therefore, functional validation of the prioritized genes and loci using *in vitro* or *in vivo* studies is required to confirm causality and further define their roles in linking aging and GCA. In addition, although our drug repurposing analysis offers promising therapeutic options, these predictions are based on *in silico* approaches and require experimental validation to assess their efficacy and safety in the context of GCA.

In conclusion, this study demonstrates for the first time a significant genetic overlap between GCA and biomarkers of biological aging. Our findings offer valuable insights into the molecular pathways linking aging and GCA, opening new avenues for research and emphasizing the potential for clinical translation, which could contribute to the development of improved treatments for GCA and other age-related diseases.

## Supplementary Materials

The Supplementary data can be found online at: www.aginganddisease.org/EN/10.14336/AD.2025.0609.



## Data Availability

Complete summary statistics of the four cross-trait meta-analyses have been deposited in the Zenodo repository (https://doi.org/10.5281/zenodo.16743049). All other data are included in the manuscript and supplementary information.
