# Supplementary material for: Cross-Trait Meta-Analysis Reveals a Genetic Link between Inflammation and Aging in Giant Cell Arteritis
Source: Aging Dis. 2026 Aug 5;17(5):2604–17. doi: 10.14336/AD.2025.0609 (PMC13437121; doi:10.14336/AD.2025.0609)
Supplement: Supplementary file 1 [file AD-17-5-2604-s.pdf]

SUPPLEMENTARY DATA

Cross-Trait Meta-Analysis Reveals a Genetic Link between Inflammation and Aging in Giant Cell Arteritis

Laura Martínez-Gutiérrez, Inmaculada Rodriguez-Martin, Gonzalo Borrego-Yaniz, Martin Kerick, International GCA Consortium, UK GCA Consortium, Carlo Salvarani, José Hernández-Rodríguez, María Cinta Cid, Miguel Ángel González-Gay, Ann W. Morgan, Javier Martín, Lourdes Ortiz-Fernández, Ana Márquez

© 2025. Martínez-Gutiérrez L et al. Published online at http://www.aginganddisease.org/EN/10.14336/AD.2025.0609

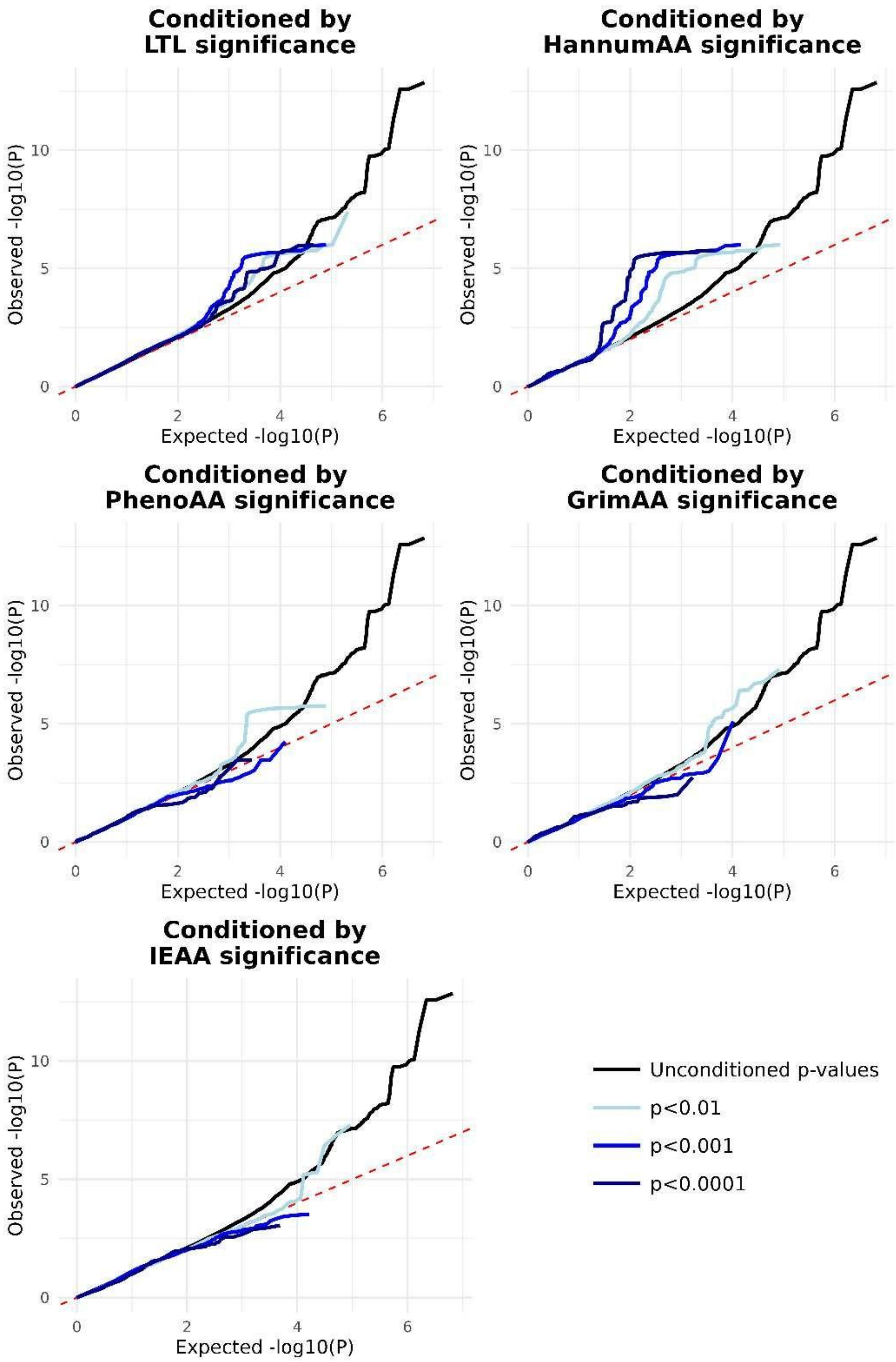

Supplementary Figure 1. Conditioned quantile-quantile (Q-Q) plots illustrating the distribution of p-values for giant cell arteritis (GCA). The black line represents all single-nucleotide polymorphisms (SNPs) without conditioning, while the colored lines represent SNPs associated with different aging markers: leukocyte telomere length (LTL), HannumAge acceleration (HannumAA), PhenoAge acceleration (PhenoAA), GrimAge acceleration (GrimAA), and intrinsic epigenetic age acceleration (IEAA), at different significance thresholds: $p < 0.01$ (light blue), $p < 0.001$ (medium blue), and $p < 0.0001$ (dark blue).

© 2025. Martínez-Gutiérrez L et al. Published online at http://www.aginganddisease.org/EN/10.14336/AD.2025.0609

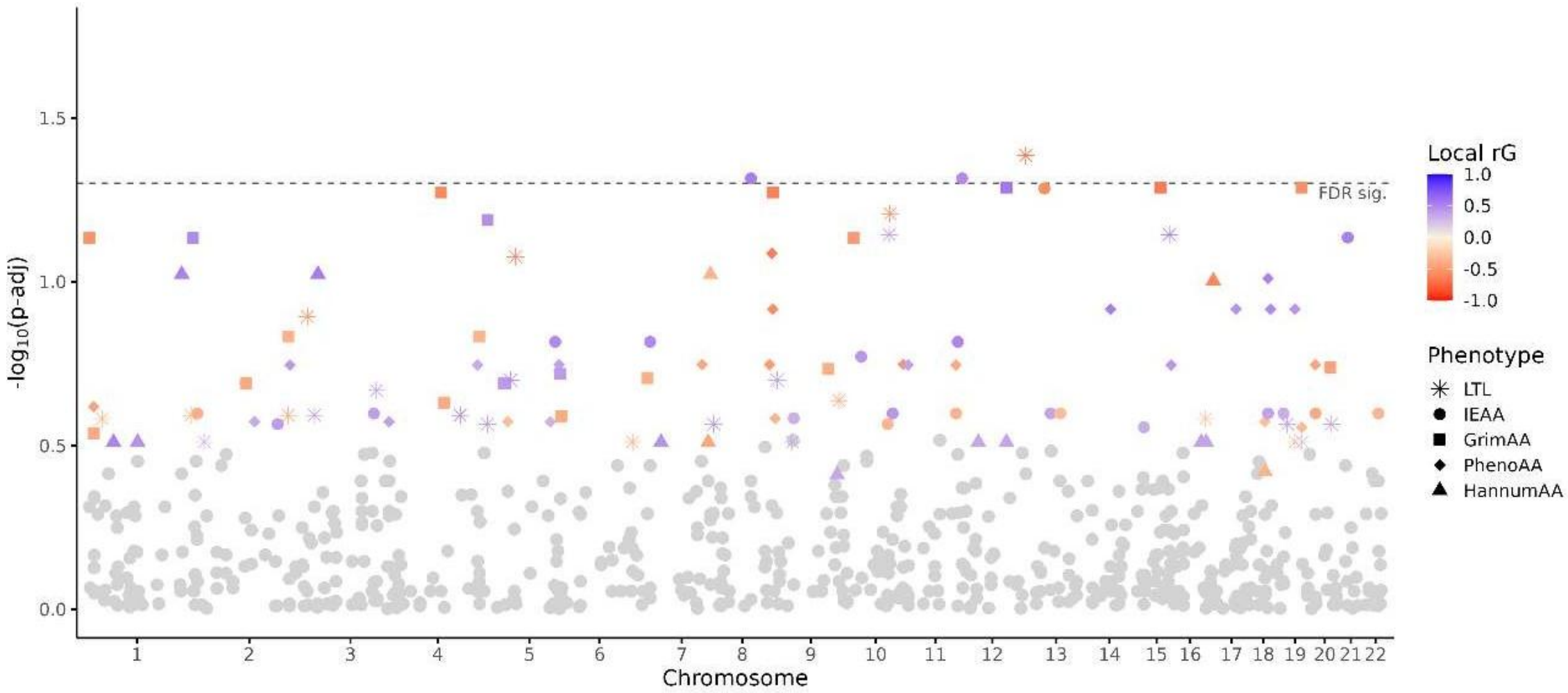

Supplementary Figure 2. Local genetic correlation between giant cell arteritis and aging markers across the genome. Each point represents a genomic region with an estimated local genetic correlation (rG) between giant cell arteritis (GCA) and one of five aging-related traits: leukocyte telomere length (LTL, asterisk), GrimAge acceleration (GrimAA, circle), PhenoAge acceleration (PhenoAA, square), intrinsic epigenetic age acceleration (IEAA, diamond), and HannumAge acceleration (HannumAA, triangle). The x-axis indicates genomic position by chromosome, and the y-axis shows the $-\log_{10}$(p-value) of the local rG estimate. Only those loci showing at least nominally significant local genetic correlation ($p < 0.05$) are color-coded according to the direction and magnitude of rG (blue for positive, red for negative). The dashed line represents FDR-adjusted p-values of 0.05.

© 2025. Martínez-Gutiérrez L et al. Published online at http://www.aginganddisease.org/EN/10.14336/AD.2025.0609

Region	Lead_SNP	Gene	Closest gene to lead	Gene_body	Exonic	Near	Blood_eQTL	Immune_eQTL	Vascular_eQTL	pQTL	sQTL	V2G
1p13.2	rs1217404	*RSBN1*										0.06
		PTPN22										0.21
		BCL2L15										0.06
		AP4B1										0.19
		DCLRE1B										0.16
2p14	rs10190233	*ACTR2*										0.09
		SPRED2										0.26
3q25.33	rs12630770	*IFT80*										0.09
		SMC4										0.28
		TRIM59										0.14
		KPNA4										0.08
		B3GALNT1										0.08
		NMD3										0.01
4p16.3	rs7666644	*POLN*										0.23
		HAUS3										0.06
		MXD4										0.10
4q24	rs230533	*BANK1*										-
		CISD2										0.07
		NFKB1										0.30
		MANBA										0.24
5q31.1	rs1981627	*SAR1B*										0.06
		JADE2										0.22
5q31.1	rs1393082	*C5orf66*										0.02
		MACROH2A1										0.08
6q21	rs12200757	*SESN1*										0.11
		AK9										0.09
		FIG4										0.05
		CD164										-
		SMPD2										-
6q26	rs11751347	*SLC22A3*										0.03
		PLG										0.18
		LPA										0.07
7q32.1	rs3807304	*CALU*										0.03
		CCDC136										0.05
		IRF5										0.25
		TNPO3										0.21
		TSPAN33										0.11
8q12.1	rs4737427	*CHCHD7*										0.20
		TGS1										0.09
		PENK										0.10
		SDR16C5										0.09
11q12.1	rs11229011	*TIMM10*										0.15
		SERPING1										0.10
		TNKS1BP1										0.15
		P2RX3										0.15
		PRG3										0.07
		PRG2										0.21
		MED19										0.03
		SMTNL1										0.04

© 2025. Martínez-Gutiérrez L et al. Published online at http://www.aginganddisease.org/EN/10.14336/AD.2025.0609

12q13.13	rs4326844	*CBX5*										0.06
		NFE2										0.17
		COPZ1										0.13
		GPR84										0.19
		SMUG1										0.11
		GTSF1										0.05
		NCKAP1L										0.13
		PDE1B										0.11
12q24.12	rs4766578	*GPN3*										-
		FAM216A										0.03
		VPS29										0.03
		PPTC7										-
		TCTN1										0.05
		HVCN1										-
		ACAD10										0.04
		TMEM116										0.09
		NAA25										0.03
		TRAFD1										0.08
		HECTD4										-
		FAM109A										0.13
		SH2B3										0.17
		ATXN2										0.07
		ALDH2										0.12
16p13.3	rs17256002	*NAA60*										0.05
		SLX4										0.07
		TRAP1										0.33
		CLUAP1										0.12
16q12.1	rs6500291	*CNEP1R1*										0.05
		TENT4B										0.15
		HEATR3										0.33
16q23.1	rs147022170	*GLG1*										0.13
		MLKL										0.08
		RFWD3										0.09
17p13.3	rs12450118	*HIC1*										0.11
		SMG6										0.08
		SRR										0.25
		TSR1										0.05
18p11.32	rs2853741	*CLUL1*										0.06
		TYMS										0.15
		ENOSF1										0.34
		THOC1										0.09
18q12.3	rs117598048	*SETBP1*										0.05
18q21.2	rs5825069	*POLI*										0.21
		C18orf54										0.15
		STARD6										0.10

Supplementary Figure 3. Functional annotation and gene prioritization for the identified pleiotropic signals. The colours indicate the SNPs that overlap with the considered functional annotations for each specific gene. Different colours indicate different categories of annotations, including the genomic location of the genetic variants, their correlation with gene expression, splicing changes, and protein levels, and their V2G score from Open Target Genetics. SNP, single-nucleotide polymorphism; eQTL, expression quantitative trait loci; sQTL, splicing quantitative trait loci; pQTL, protein quantitative trait loci; V2G, Variant-to-Gene.

© 2025. Martínez-Gutiérrez L et al. Published online at http://www.aginganddisease.org/EN/10.14336/AD.2025.0609

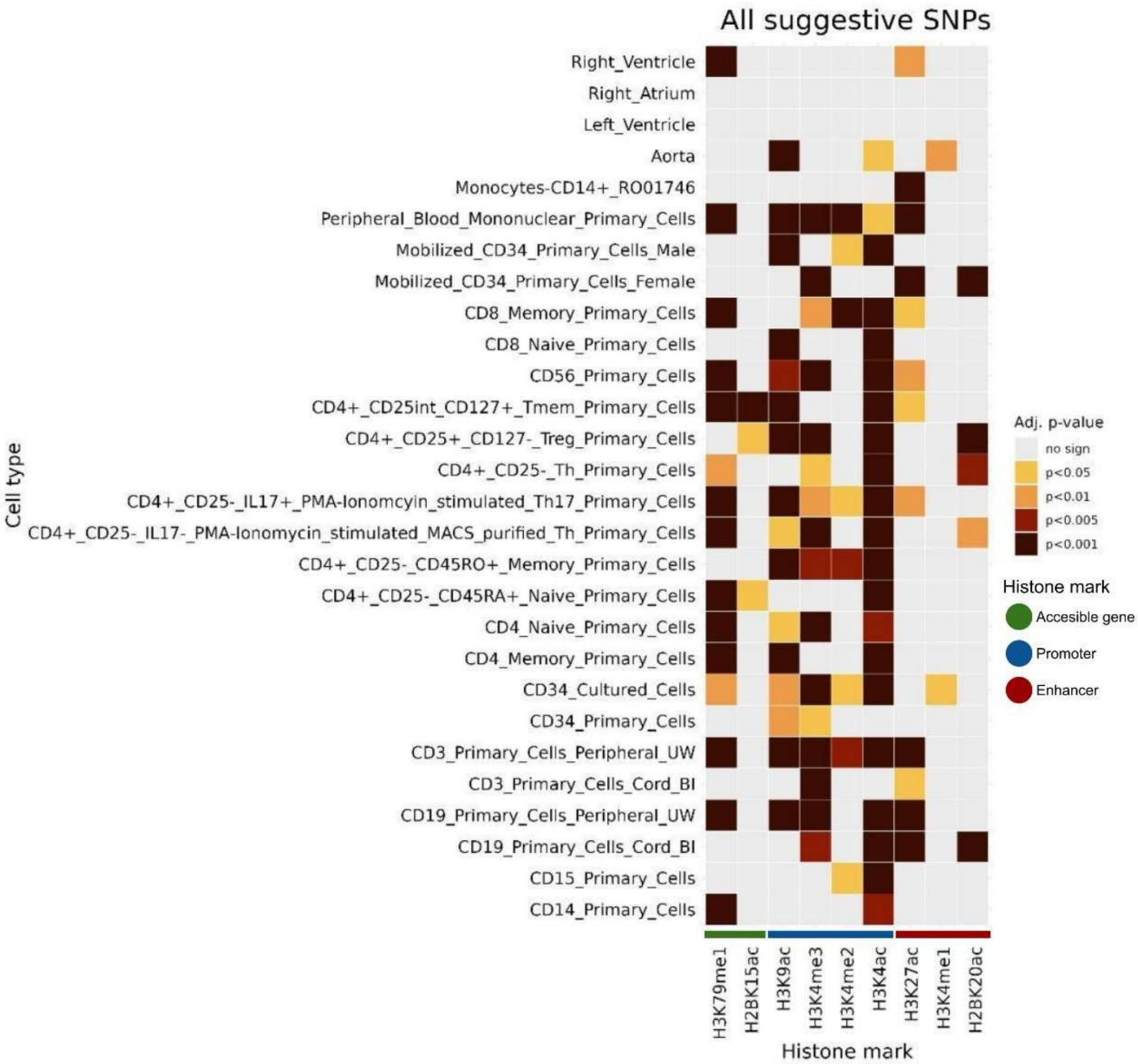

Supplementary Figure 4. Results of the histone mark enrichment analysis for all suggestive pleiotropic variants between giant cell arteritis (GCA) and aging markers. The analysis was performed using GoShifter, testing for enrichment of these variants in genomic regions marked by histone modifications across various blood, immune, and vascular cell types. The y- axis lists the analysed cell types, while the x-axis represents histone modifications associated with accessible genes (green), promoters (blue), and enhancers (red). Each cell in the heatmap represents the statistical significance of enrichment for the given histone mark in the corresponding cell type, with color intensity indicating adjusted p-values (darker shades represent stronger significance). Histone mark data were obtained from the Roadmap Epigenomics project. Adjusted p-values were calculated using FDR correction.

© 2025. Martínez-Gutiérrez L et al. Published online at http://www.aginganddisease.org/EN/10.14336/AD.2025.0609

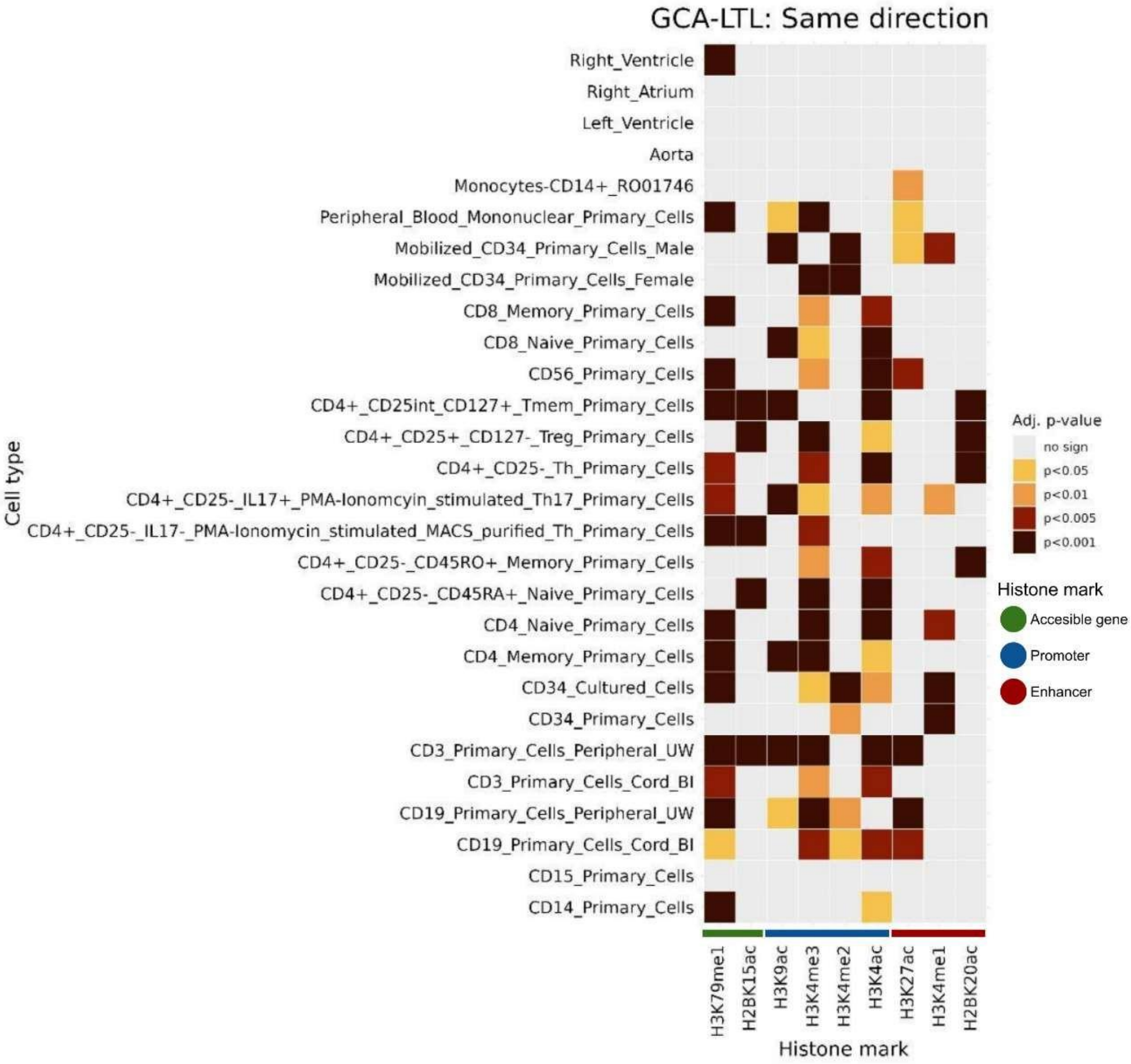

Supplementary Figure 5. Results of the histone mark enrichment analysis for genetic variants showing concordant effects between giant cell arteritis (GCA) and leukocyte telomere length (LTL). Concordant variants were defined as those associated with GCA and LTL, showing the same direction of effect. The analysis was performed using GoShifter, testing for enrichment of these variants in genomic regions marked by histone modifications across various blood, immune, and vascular cell types. The y-axis lists the analysed cell types, while the x-axis represents histone modifications associated with accessible genes (green), promoters (blue), and enhancers (red). Each cell in the heatmap represents the statistical significance of enrichment for the given histone mark in the corresponding cell type, with color intensity indicating adjusted p-values (darker shades represent stronger significance). Histone mark data were obtained from the Roadmap Epigenomics project. Adjusted p-values were calculated using FDR correction.

© 2025. Martínez-Gutiérrez L et al. Published online at http://www.aginganddisease.org/EN/10.14336/AD.2025.0609

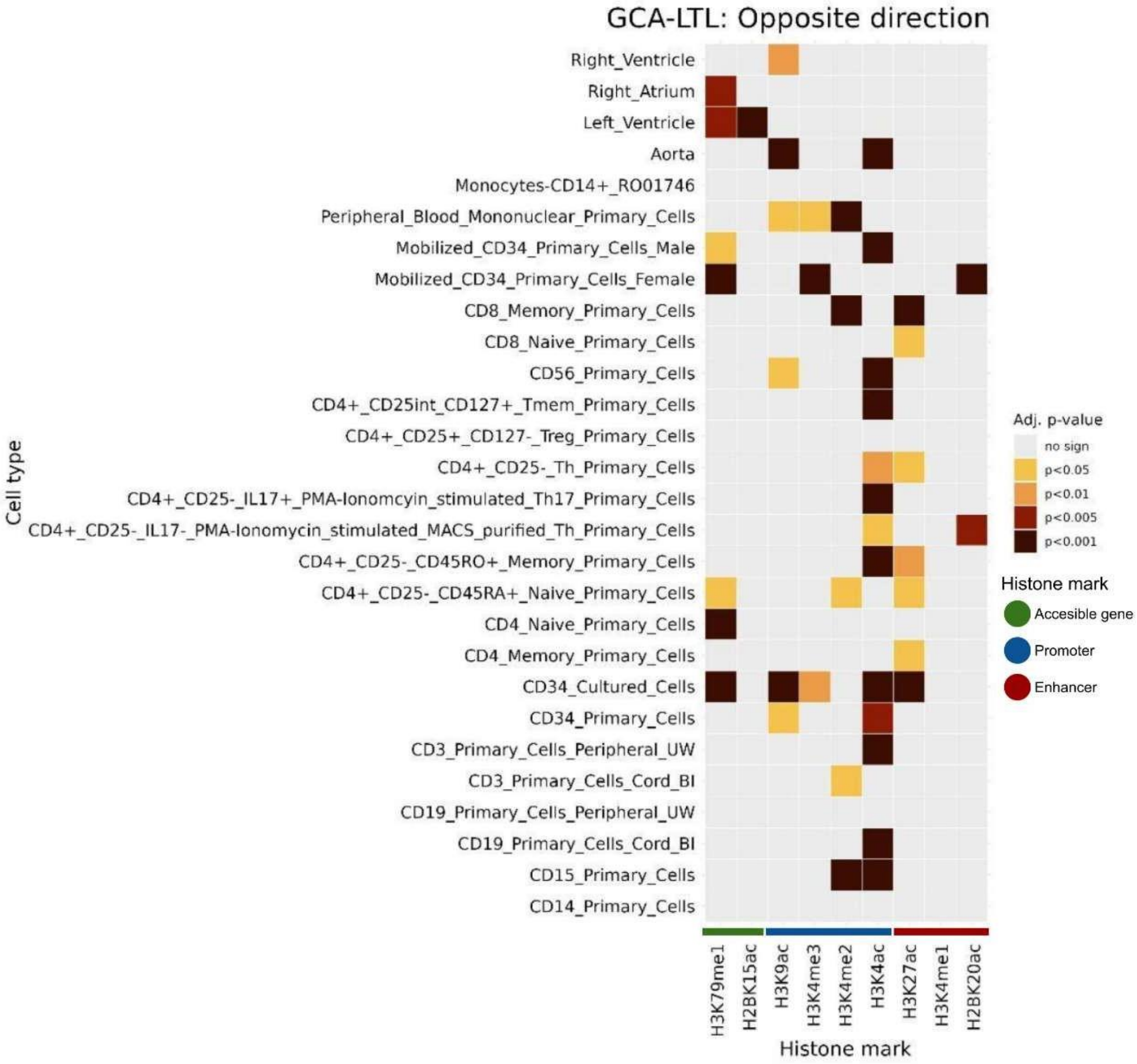

Supplementary Figure 6. Results of the histone mark enrichment analysis for genetic variants showing discordant effects between giant cell arteritis (GCA) and leukocyte telomere length (LTL). Discordant variants were defined as those associated with GCA and LTL, showing opposite direction of effect. The analysis was performed using GoShifter, testing for enrichment of these variants in genomic regions marked by histone modifications across various blood, immune, and vascular cell types. The y-axis lists the analysed cell types, while the x-axis represents histone modifications associated with accessible genes (green), promoters (blue), and enhancers (red). Each cell in the heatmap represents the statistical significance of enrichment for the given histone mark in the corresponding cell type, with color intensity indicating adjusted p-values (darker shades represent stronger significance). Histone mark data were obtained from the Roadmap Epigenomics project. Adjusted p-values were calculated using FDR correction.

© 2025. Martínez-Gutiérrez L et al. Published online at http://www.aginganddisease.org/EN/10.14336/AD.2025.0609

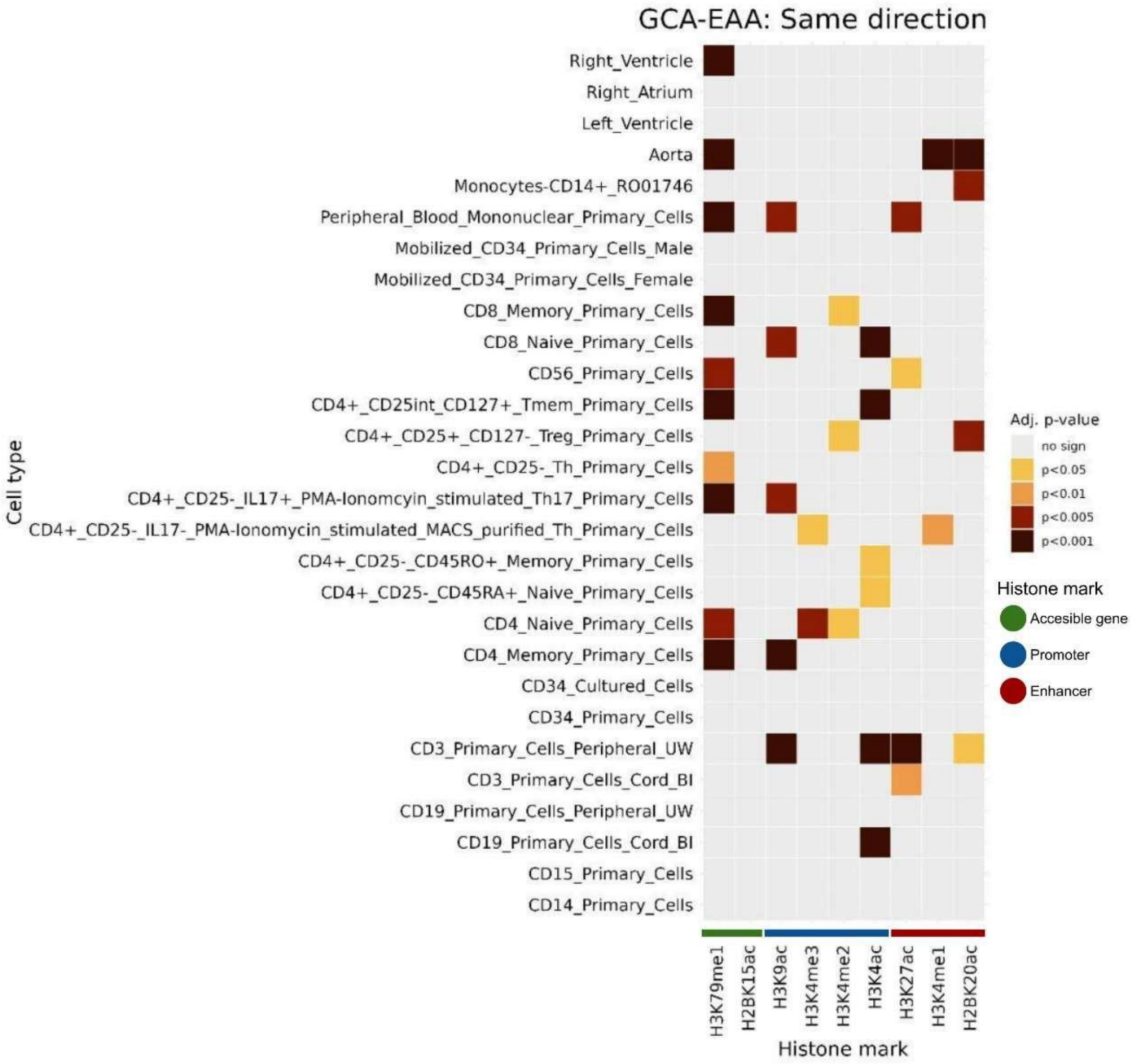

Supplementary Figure 7. Results of the histone mark enrichment analysis for genetic variants showing concordant effects between giant cell arteritis (GCA) and epigenetic age acceleration (EAA). Concordant variants were defined as those associated with GCA and EAA, showing the same direction of effect. The analysis was performed using GoShifter, testing for enrichment of these variants in genomic regions marked by histone modifications across various blood, immune, and vascular cell types. The y-axis lists the analysed cell types, while the x-axis represents histone modifications associated with accessible genes (green), promoters (blue), and enhancers (red). Each cell in the heatmap represents the statistical significance of enrichment for the given histone mark in the corresponding cell type, with color intensity indicating adjusted p-values (darker shades represent stronger significance). Histone mark data were obtained from the Roadmap Epigenomics project. Adjusted p-values were calculated using FDR correction.

© 2025. Martínez-Gutiérrez L et al. Published online at http://www.aginganddisease.org/EN/10.14336/AD.2025.0609

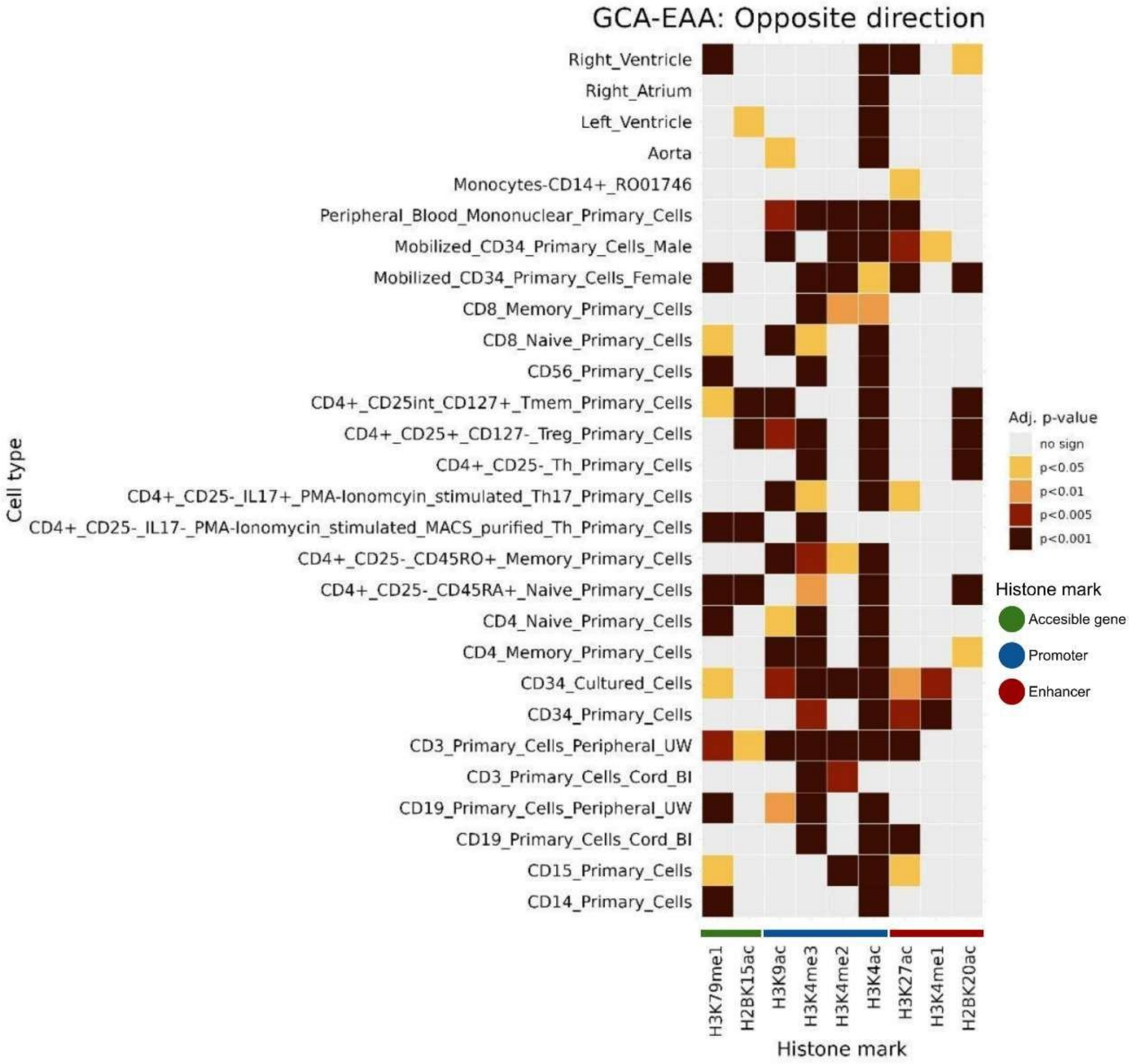

Supplementary Figure 8. Results of the histone mark enrichment analysis for genetic variants showing discordant effects between giant cell arteritis (GCA) and epigenetic age acceleration (EAA). Discordant variants were defined as those associated with GCA and EAA, showing opposite direction of effect. The analysis was performed using GoShifter, testing for enrichment of these variants in genomic regions marked by histone modifications across various blood, immune, and vascular cell types. The y-axis lists the analysed cell types, while the x-axis represents histone modifications associated with accessible genes (green), promoters (blue), and enhancers (red). Each cell in the heatmap represents the statistical significance of enrichment for the given histone mark in the corresponding cell type, with color intensity indicating adjusted p-values (darker shades represent stronger significance). Histone mark data were obtained from the Roadmap Epigenomics project. Adjusted p-values were calculated using FDR correction.

© 2025. Martínez-Gutiérrez L et al. Published online at http://www.aginganddisease.org/EN/10.14336/AD.2025.0609

1p13.2

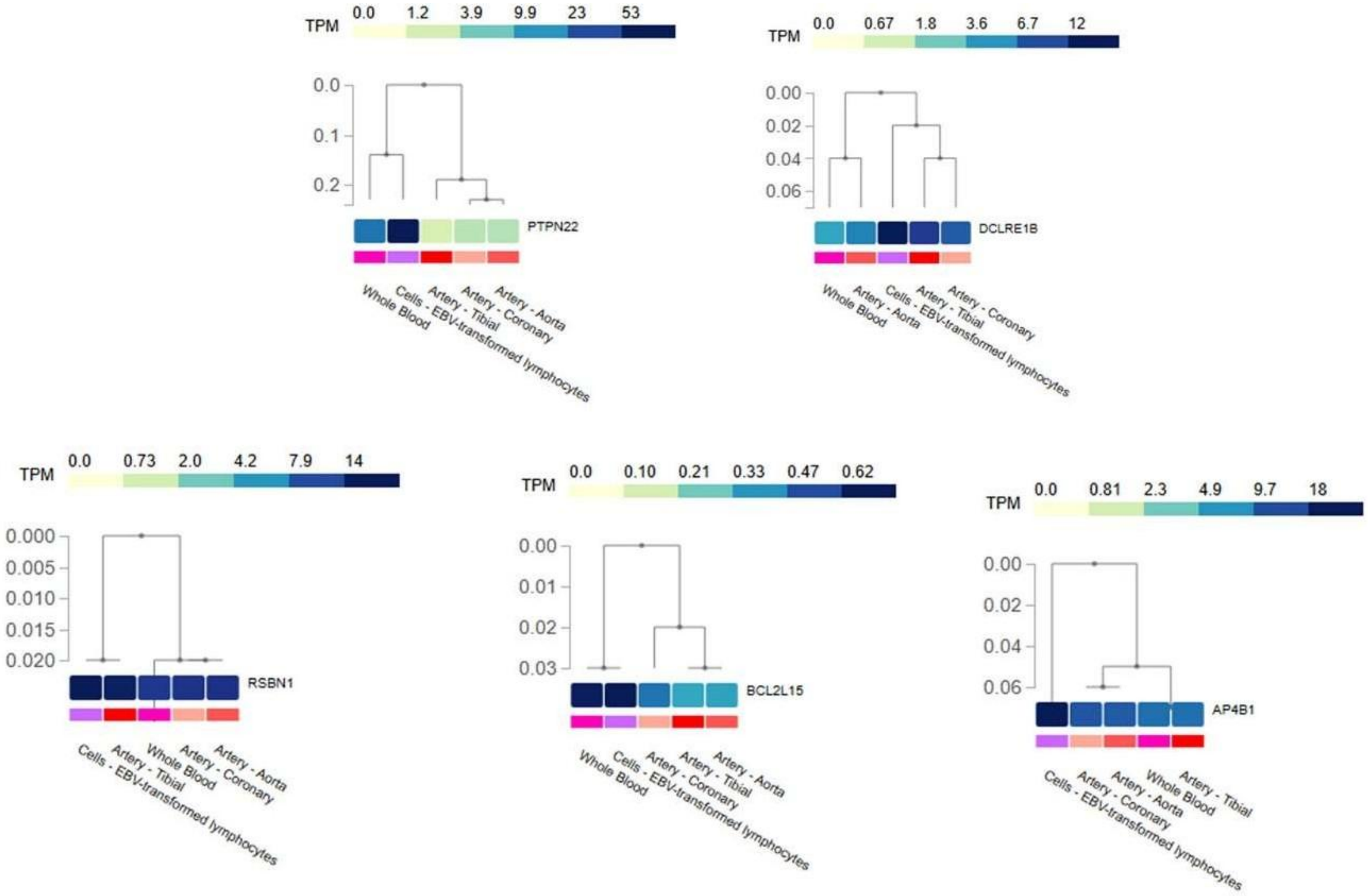

2p14

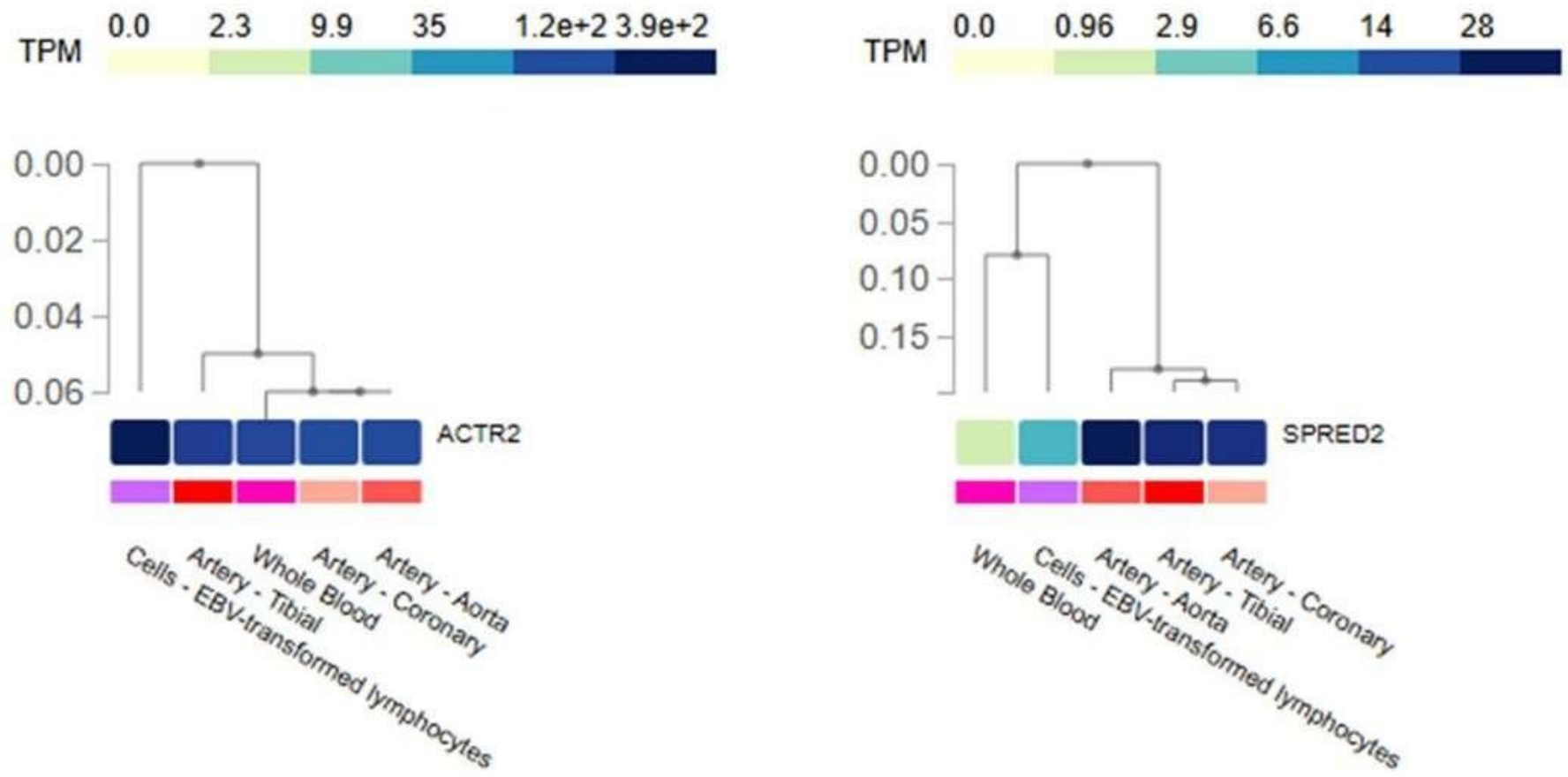

© 2025. Martínez-Gutiérrez L et al. Published online at http://www.aginganddisease.org/EN/10.14336/AD.2025.0609

4q24

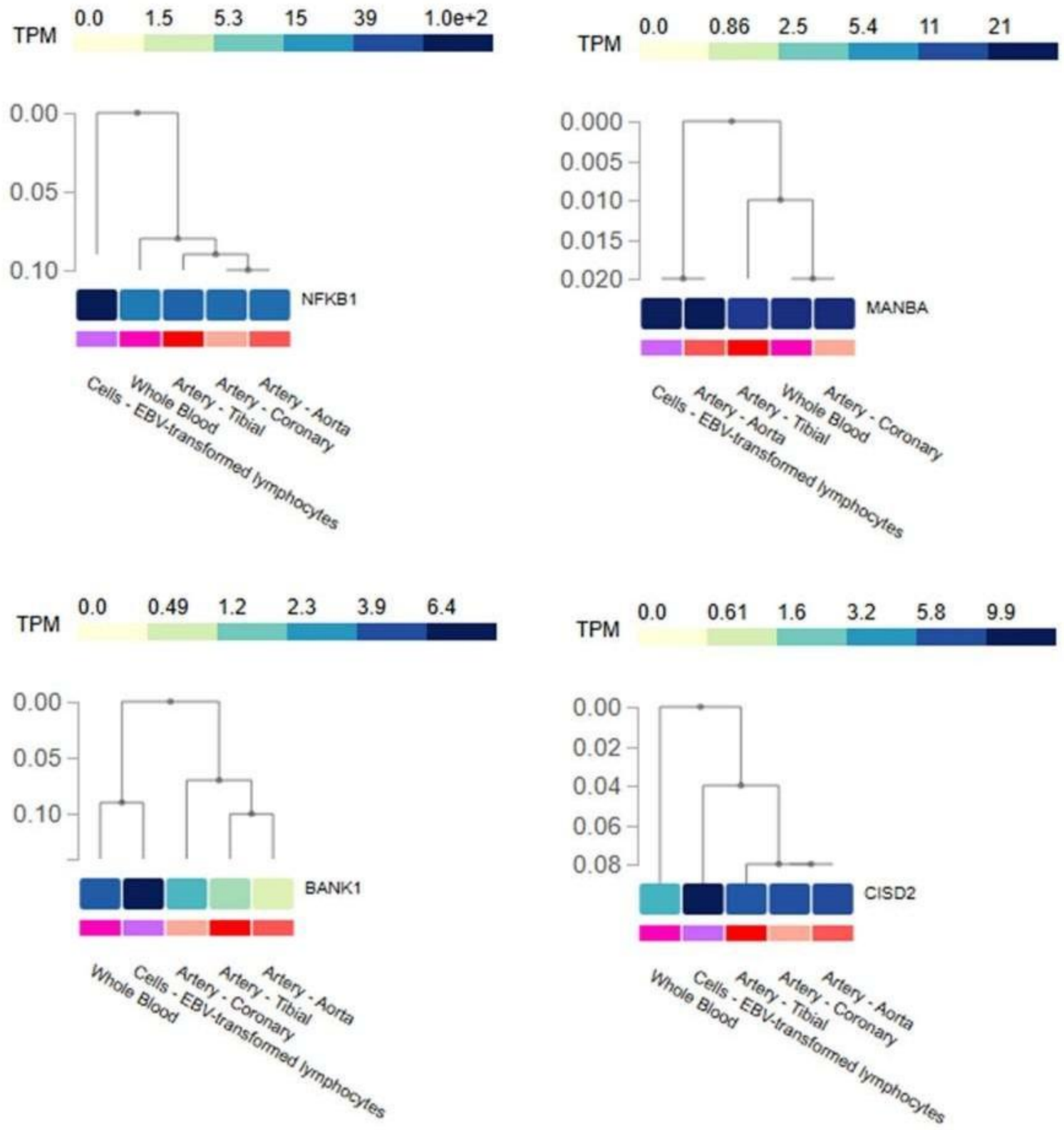

6q26

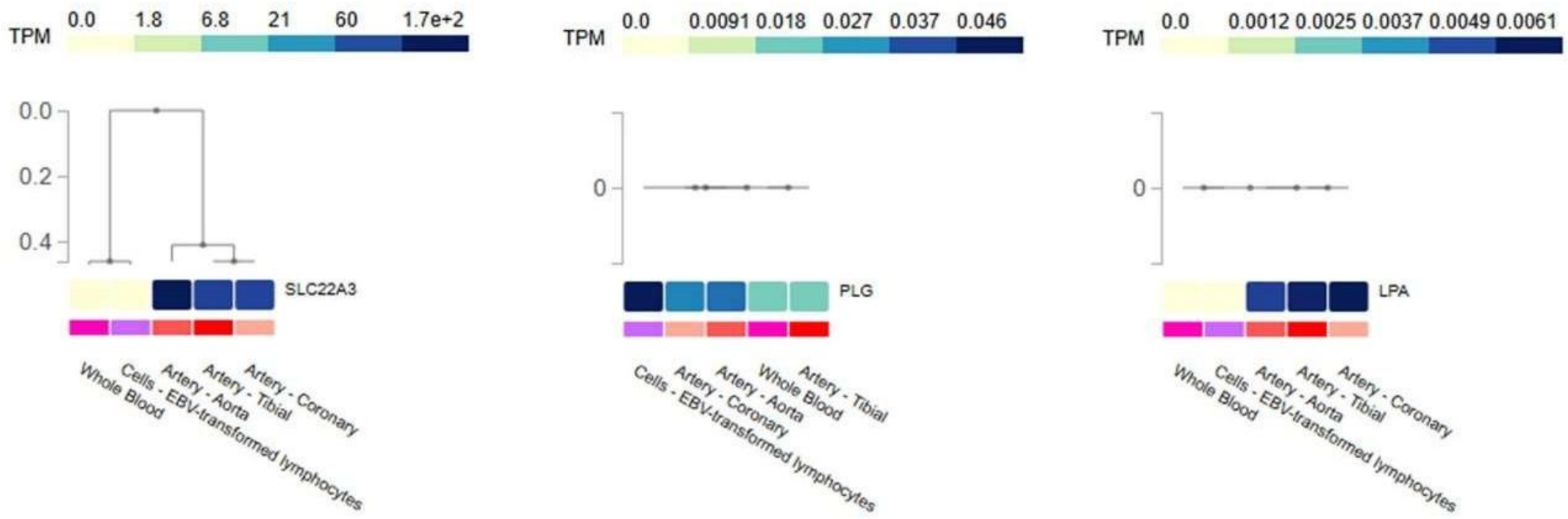

© 2025. Martínez-Gutiérrez L et al. Published online at http://www.aginganddisease.org/EN/10.14336/AD.2025.0609

7q32.1

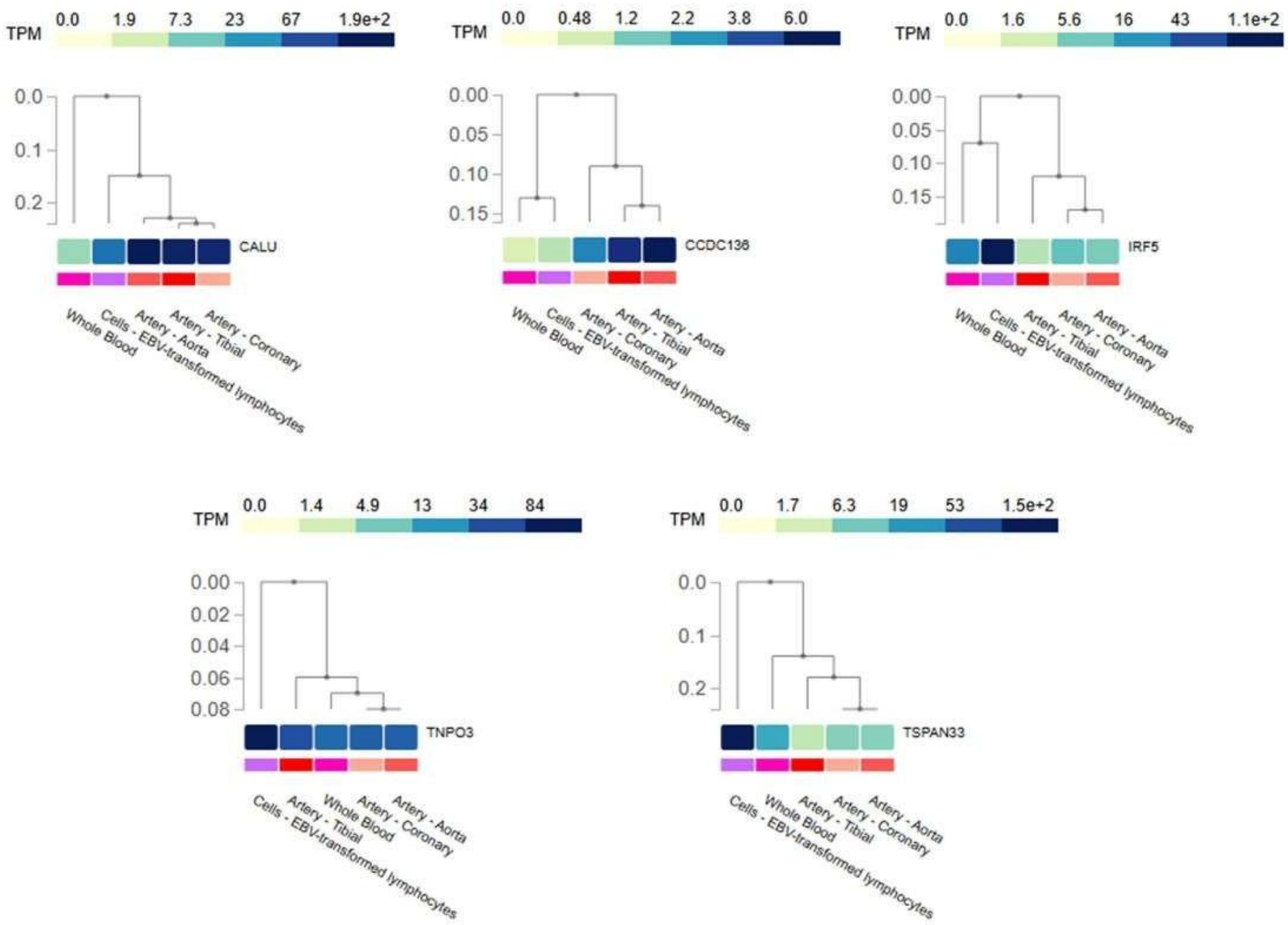

8q12.1

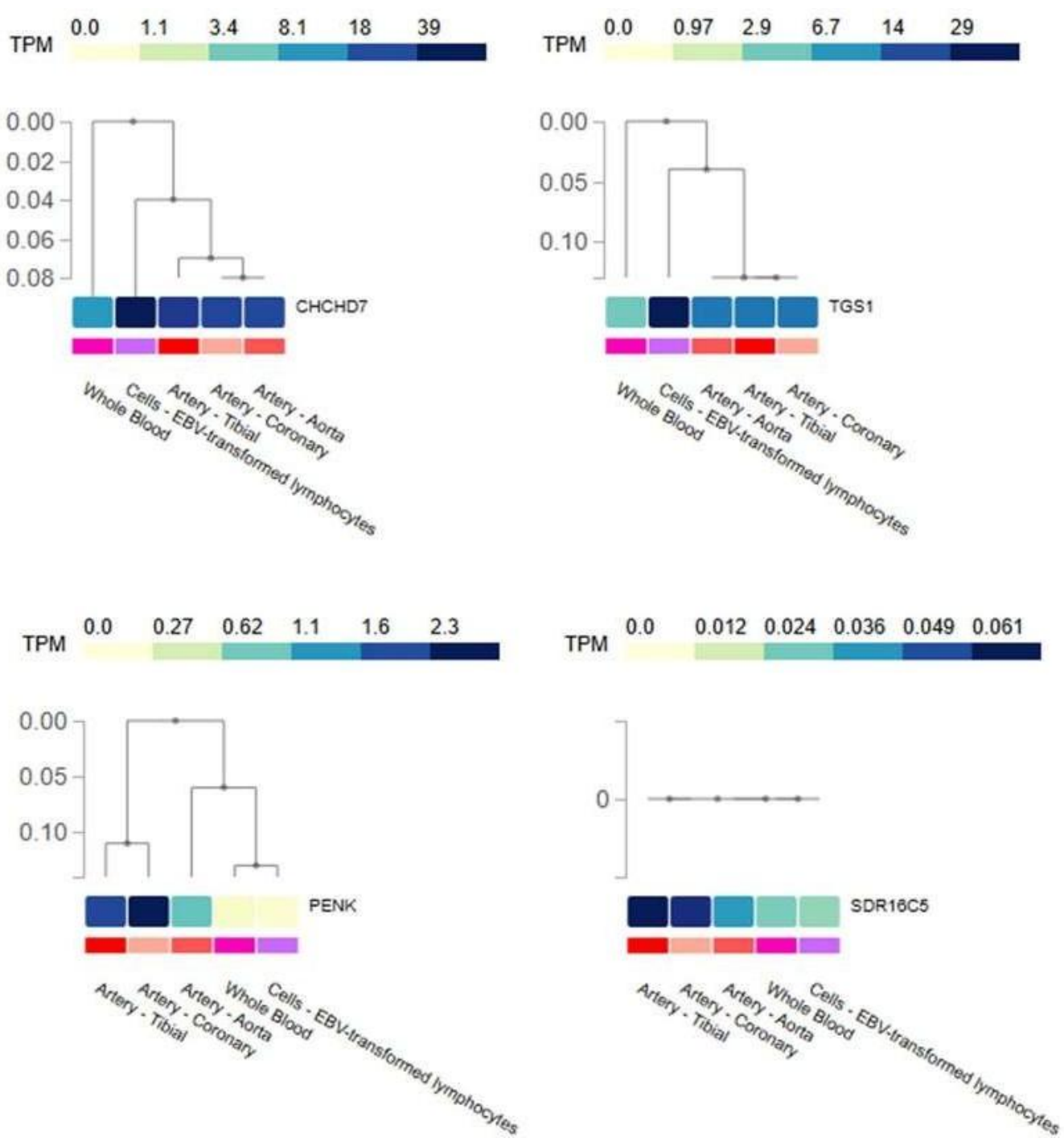

© 2025. Martínez-Gutiérrez L et al. Published online at http://www.aginganddisease.org/EN/10.14336/AD.2025.0609

16q23.1

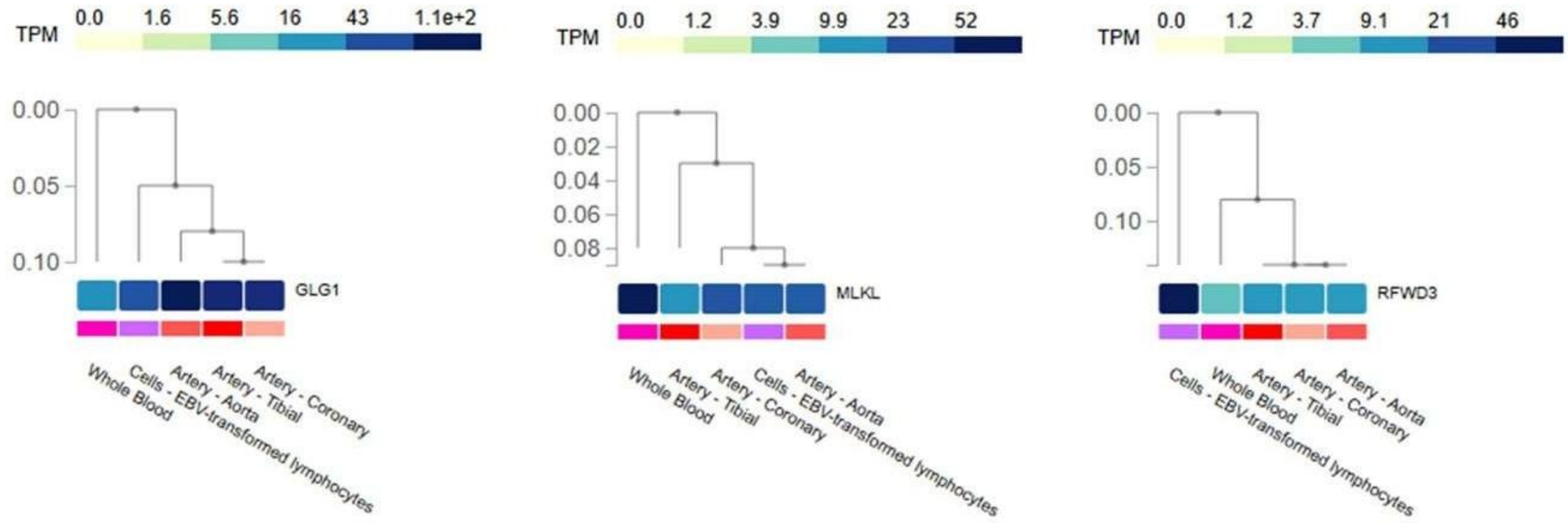

17p13.3

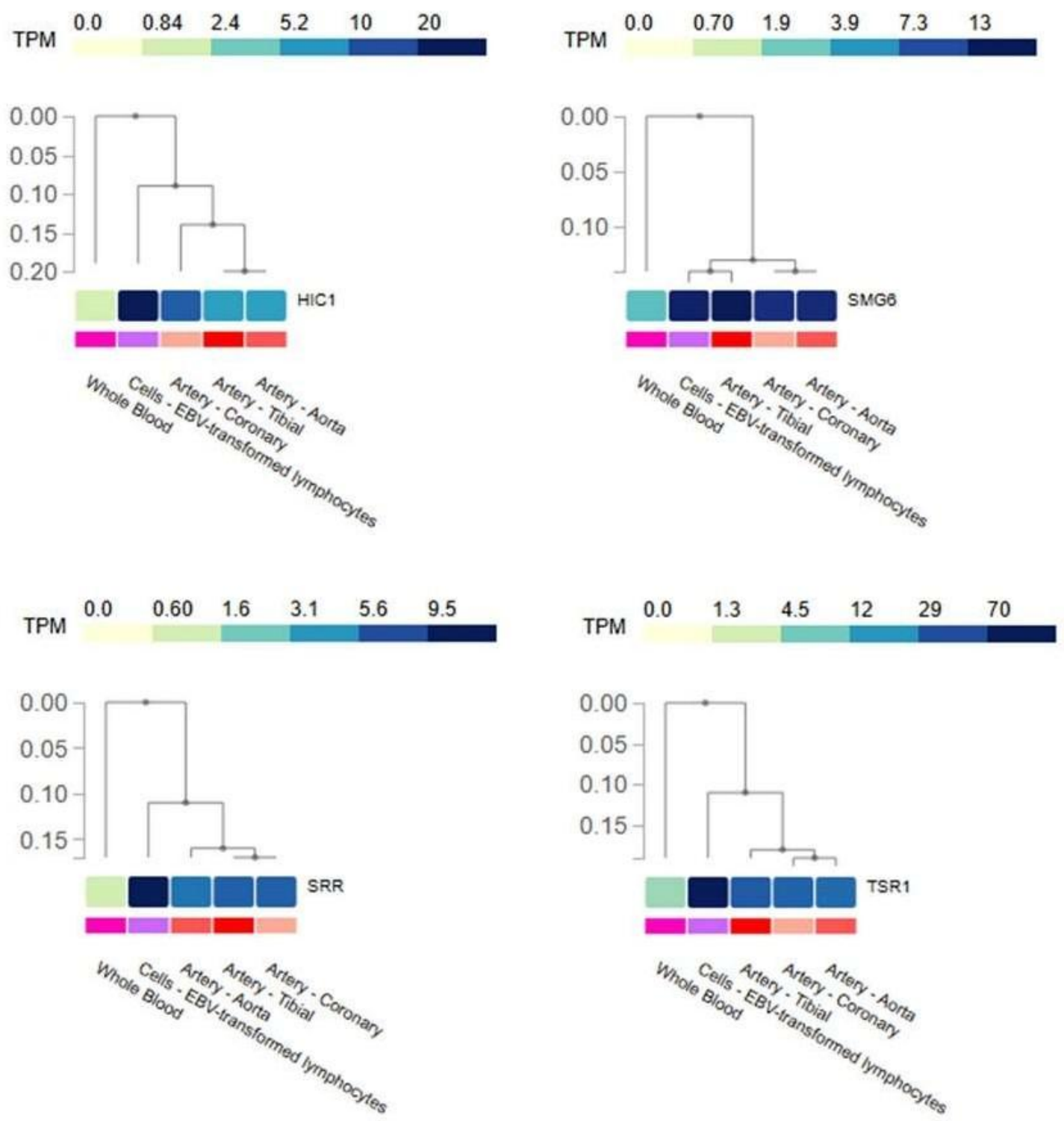

Supplementary Figure 9. Tissue-specific expression of prioritized genes within loci showing opposite genetic effects between giant cell arteritis (GCA) and aging markers. Heatmaps display transcript per million (TPM) expression levels across selected human tissues relevant to GCA pathogenesis. Color intensity reflects expression magnitude, with darker shades indicating higher expression.

© 2025. Martínez-Gutiérrez L et al. Published online at http://www.aginganddisease.org/EN/10.14336/AD.2025.0609

SUPPLEMENTARY DATA

Supplementary Table 1. GWAS summary data used for cross-trait meta-analysis

Trait	N	Cases	Controls	Original number of SNPs	Analyzed number of SNPs	PMID ID
**GCA**	19,048	3,498	15,550	6,691,294	6,691,295	38734017
**LTL**	472,174	-	-	20,134,421	6,641,825	34611362
**HannumAA**	34,710	-	-	7,565,494	6,168,770	34187551
**IEAA**	34,710	-	-	7,567,981	6,168,919	34187551
**PhenoAA**	34,710	-	-	7,568,034	6,168,917	34187551
**GrimAA**	34,710	-	-	7,568,150	6,168,940	34187551

GCA, Giant Cell Arteritis; LTL, Leukocyte Telomere Length; HannumAA, HannumAge Acceleration; IEAA, Intrinsic Epigenetic Age Acceleration; PhenoAA, PhenoAge Acceleration; GrimAA, GrimAge Acceleration; SNP, single-nucleotide polymorphism.

Supplementary Table 2. FDR-significant local genetic correlations between giant cell arteritis and aging markers.

Locus	chr:pos (hg19)	N snps	N PCs	Phenotype1	Phenotype2	Local rG	P-value	Adjusted p-value
1862	chr12:132957946-133841814	1285	96	GCA	LTL	-0,68 (-1 - -0,37)	2.62E-04	4.11E-02
1733	chr11:128262551-129451784	2105	242	GCA	IEAA	0,49 (0,22 - 0,78)	6.53E-04	4.83E-02
1320	chr8:87563146-89081177	3170	232	GCA	IEAA	0,55 (0,24 - 0,91)	7.55E-04	4.83E-02

GCA, Giant Cell Arteritis; LTL, Leukocyte Telomere Length; IEAA, Intrinsic Epigenetic Age Acceleration; N SNP, number of single-nucleotide polymorphism; N PCs, number of principal components; rG, genetic correlation.

© 2025. Martínez-Gutiérrez L et al. Published online at http://www.aginganddisease.org/EN/10.14336/AD.2025.0609

Supplementary Table 3. Independent genetic variants reaching genome-wide significant level in the subset-based meta-analysis. Results of the subset-based meta-analysis and of each independent trait are shown.

			Subset-based meta-analysis			GCA		LTL		GrimAA		IEAA		HannumAA		PhenoAA	
**SNP**	**Nearest gene**	**EA**	**p-value**	**OR (95% CI)**	**Best subest**	**p-value**	**OR**	**p-value**	**OR**	**p-value**	**OR**	**p-value**	**OR**	**p-value**	**OR**	**p-value**	**OR**
rs1217404	*PTPN22*	T	3.97E-14	1.08 (1.02-1.15) | 0.99 (0.98-0.99)	GCA | LTL IEAA HannumAA GrimAA PhenoAA	7.05E-03	1.08	1.20E-13	0.99	2.07E-01	0.96	1.99E-01	0.96	2.05E-01	0.96	3.10E-01	0.96
rs10190233	*SPRED2*	A	1.29E-08	1.01 (1.01-1.02) | 0.92 (0.86-0.98)	LTL GrimAA | GCA	5.82E-03	0.92	7.70E-08	1.01	2.67E-01	1.04	7.57E-01	1.01	1.94E-01	0.96	9.29E-01	1.00
rs12630770	*IFT80*	A	4.23E-16	1.17 (1.09-1.25)| 0.98 (0.98-0.99)	IEAA HannumAA GrimAA PhenoAA | GCA LTL	9.27E-03	0.92	8.40E-12	0.98	4.13E-02	1.07	7.97E-06	1.17	1.01E-02	1.09	9.60E-04	1.16
rs7666644	*POLN*	T	1.78E-14	1.16 (1.04-1.30) | 0.98 (0.97-0.98)	GCA | LTL HannumAA	9.36E-03	1.16	1.50E-13	0.98	8.96E-01	0.99	5.69E-01	0.97	4.78E-02	0.91	8.58E-01	1.01
rs230533	*NFKB1*	A	7.03E-10	1.16 (1.09-1.23) | 0.87 (0.81-0.92)	GCA | HannumAA	2.44E-06	1.16	3.10E-04	1.01	4.06E-01	1.03	7.17E-01	0.99	5.82E-06	0.87	1.09E-02	1.11
rs1981627	*JADE2*	A	5.39E-09	1.15 (1.08-1.22) | 0.99 (0.99-1.00)	IEAA | GCA LTL	2.79E-03	0.91	1.30E-04	0.99	2.00E-03	1.10	7.86E-06	1.15	1.93E-03	1.10	6.32E-02	1.07
rs1393082	*MACROH2A1*	A	2.47E-09	1.16 (1.10-1.22) | 0.99 (0.99-1.00)	GCA IEAA | LTL	1.74E-03	1.13	8.30E-03	0.99	1.91E-01	1.04	6.21E-07	1.18	1.02E-02	1.09	2.24E-03	1.14
rs12200757	*CD164*	T	1.26E-10	1.01 (1.01-1.02) | 0.89 (0.83-0.95)	GCA LTL | GrimAA	5.44E-03	1.11	2.70E-08	1.01	1.04E-03	0.89	9.98E-01	1.00	1.09E-01	0.95	8.25E-01	0.99
rs11751347	*LPA*	T	2.43E-08	1.13 (1.02-1.25) | 0.74 (0.66-0.82)	HannumAA | GCA	2.64E-08	0.73	1.20E-02	0.99	1.62E-01	1.08	1.23E-01	1.09	2.02E-02	1.13	1.29E-01	1.11
rs3807304	*TNPO3*	T	1.16E-09	1.02 (1.01-1.02) | 0.88 (0.82-0.96)	LTL IEAA | GCA	2.42E-03	0.88	2.10E-08	1.02	4.63E-01	1.03	1.74E-01	1.06	9.85E-01	1.00	7.54E-01	1.02
rs4737427	*SDR16C5*	A	4.41E-09	1.02 (1.01-1.02) | 0.87 (0.79-0.96)	LTL PhenoAA | GCA	4.80E-03	0.87	2.30E-08	1.02	8.19E-01	1.01	7.17E-01	1.02	9.76E-01	1.00	3.41E-01	1.05
rs11229011	*P2RX3*	A	1.20E-08	1.18 (1.12-1.25)	GCA IEAA	7.57E-03	1.11	6.80E-01	1.00	4.09E-01	1.04	3.98E-08	1.27	5.99E-01	0.98	7.42E-01	0.98
rs4326844	*COPZ1*	A	1.08E-08	1.11 (1.05-1.17) | 0.99 (0.99-0.99)	GCA PhenoAA | LTL	5.87E-03	1.10	2.70E-06	0.99	1.95E-01	1.04	2.87E-01	1.03	7.47E-02	1.06	3.38E-03	1.12
rs4766578	*ATXN2*	A	3.92E-17	1.02 (1.01-1.02) | 0.91 (0.84-0.98)	GCA LTL | PhenoAA	2.31E-03	1.15	1.70E-15	1.02	1.77E-01	0.96	3.54E-01	0.97	3.50E-01	1.03	8.67E-03	0.91
rs17256002	*TRAP1*	A	9.98E-09	1.18 (1.05-1.31) | 0.98 (0.97-0.99)	GCA GrimAA | LTL	9.95E-03	1.21	1.10E-07	0.98	4.28E-02	1.15	7.41E-01	1.02	6.55E-01	1.03	9.99E-01	1.00
rs6500291	*HEATR3*	C	2.68E-11	1.01 (1.01-1.02) | 0.91 (0.85-0.96)	LTL |GCA GrimAA PhenoAA	6.56E-03	0.91	9.00E-10	1.01	1.34E-01	0.95	3.99E-01	0.97	5.01E-01	0.98	1.67E-02	0.90
rs147022170	*GLG1*	C	2.82E-14	1.03 (1.02-1.04) | 0.83 (0.74-0.95)	LTL | GCA	4.66E-03	0.83	1.70E-13	1.03								
rs12450118	*SMG6*	C	4.03E-09	1.12 (1.06-1.18) | 0.99 (0.99-0.99)	GCA | LTL IEAA HannumAA GrimAA PhenoAA	1.67E-04	1.12	2.80E-06	0.99	2.92E-01	0.97	4.60E-02	0.94	1.63E-01	0.96	2.92E-01	0.96

© 2025. Martínez-Gutiérrez L et al. Published online at http://www.aginganddisease.org/EN/10.14336/AD.2025.0609

rs2853741	*TYMS*	T	4.61E-10	0.99 (0.98-0.99)	GCA LTL HannumAA GrimAA PhenoAA IEAA	5.03E-03	0.90	5.60E-09	0.99	1.10E-01	0.95	2.95E-01	0.97	6.67E-02	0.94	6.89E-02	0.93
rs117598048	*SETBP1*	G	3.41E-17	1.04 (1.03-1.05) | 0.50 (0.38-0.65)	GCA LTL | IEAA GrimAA PhenoAA	8.67E-03	1.29	2.90E-11	1.04	1.68E-02	0.79	2.12E-03	0.73	4.70E-01	0.93	1.73E-07	0.50
rs5825069	*STARD6*	A	3.34E-17	1.02 (1.01-1.02)	GCA LTL	3.68E-03	1.11	3.50E-16	1.02								

GCA, Giant Cell Arteritis; LTL, Leukocyte Telomere Length; HannumAA, HannumAge Acceleration; IEAA, Intrinsic Epigenetic Age Acceleration; PhenoAA, PhenoAge Acceleration; GrimAA, GrimAge Acceleration; SNP, single-nucleotide polymorphism; EA, effect allele; OR, odds ratio.

© 2025. Martínez-Gutiérrez L et al. Published online at http://www.aginganddisease.org/EN/10.14336/AD.2025.0609

Supplementary Table 4. Genetic variants included in the original and revised version of the polygenic risk score for GCA prediction.

SNP	Original model		Revised model	
	Effect allele	Beta	Effect allele	Beta
rs17882084	A	0.7015	A	0.7015
rs1049087	A	0.4019	A	0.4019
rs2596501	G	0.1837	G	0.1837
rs2856726	A	0.1567	A	0.1567
HLA-DPB1*03	Present	-0.2585	Present	-0.2585
rs45485198	C	0.2607	C	0.2607
rs7772437	A	-0.181	A	-0.1810
rs7827332	G	-0.231	G	-0.2310
rs704	A	-0.1807	A	-0.1807
rs11751347	T	-0.3087	T	-0.3087
rs7090203	T	0.3365	T	0.3365
rs1217404	-	-	T	0.0334
rs10190233	-	-	A	-0.0362
rs12630770	-	-	A	-0.0088
rs7666644*	-	-	T	0.0645
rs230533	-	-	A	0.0645
rs1981627	-	-	A	-0.0044
rs1393082*	-	-	A	0.0645
rs12200757*	-	-	T	0.0043
rs11751347**	-	-	-	-
rs3807304	-	-	T	-0.0555
rs4737427*	-	-	A	-0.0605
rs11229011	-	-	A	0.0719
rs4326844*	-	-	A	0.0453
rs4766578	-	-	A	0.0086
rs17256002*	-	-	A	0.0719
rs6500291	-	-	C	-0.0410
rs147022170	-	-	C	-0.0809
rs12450118	-	-	C	0.0492
rs2853741	-	-	T	-0.0044
rs117598048*	-	-	G	0.0170
rs5825069*	-	-	A	0.0086

*The effect of these SNPs over the prediction ability of the model could not be evaluated, as they were not genotyped in the testing cohort.

** This lead SNP was already included in the original model.

SNP, single nucleotide polymorphism.

Supplementary Table 5. Expression quantitative trait loci (eQTL) effects of lead polymorphisms and their proxies (r2≥0.9) in tissues relevant to giant cell arteritis.

See: https://zenodo.org/records/16743049

© 2025. Martínez-Gutiérrez L et al. Published online at http://www.aginganddisease.org/EN/10.14336/AD.2025.0609

Supplementary Table 6. Splicing quantitative trait loci (sQTL) effects of lead polymorphisms in tissues relevant to giant cell arteritis.

Region	Lead SNP	Gene	β	P value	Database	Tissue / Cell type
1p13.2	rs1217404	*DCLRE1B*	0.343	9.30E-10	GTEx v8 sQTL datasets	Cultured fibroblasts
2p14	rs10190233	*SPRED2*	-0.424	3.20E-09	GTEx v8 sQTL datasets	Artery aorta
			-0.389	5.60E-13		Artery tibial
3q25.33	rs12630770	*SMC4*	-0.453	2.60E-06	GTEx v8 sQTL datasets	Artery coronary
			-0.291	2.30E-07		Artery tibial
			-0.336	2.10E-09		Cultured fibroblasts
			0.854	7.20E-08		EBV-transformed lymphocytes
4p16.3	rs7666644	*POLN*	-0.565	1.20E-06	GTEx v8 sQTL datasets	Cultured fibroblasts
4q24	rs230533	*NFKB1*	0.435	2.40E-09	GTEx v8 sQTL datasets	Cultured fibroblasts
5q31.1	rs1981627	*JADE2*	0.335	4.40E-08	GTEx v8 sQTL datasets	Artery tibial
16p13.3	rs17256002	*TRAP1*	1.32	1.00E-23	GTEx v8 sQTL datasets	Artery aorta
			1.61	5.60E-39	GTEx v8 sQTL datasets	Artery tibial
16q12.1	rs6500291	*HEATR3*	0.412	7.20E-07	GTEx v8 sQTL datasets	Artery aorta
			0.427	7.50E-11	GTEx v8 sQTL datasets	Artery tibial
			-0.36	2.20E-09	GTEx v8 sQTL datasets	Cultured fibroblasts
18p11.32	rs2853741	*ENOSF1*	0.491	4.20E-14	GTEx v8 sQTL datasets	Artery aorta
			0.334	1.70E-07	GTEx v8 sQTL datasets	Artery tibial
			0.307	5.20E-10	GTEx v8 sQTL datasets	Cultured fibroblasts
		THOC1	0.353	2.50E-06	GTEx v8 sQTL datasets	Cultured fibroblasts

Supplementary Table 7. Protein quantitative trait loci (pQTL) effects of lead polymorphisms.

Region	Lead SNP	Gene	β	P value	Study	Tissue / Cell type
4q24	rs230533	*MANBA*	-0.18	4.10E-12	Nature. 2018;558(7708):73–79	Blood plasma
6q26	rs11751347	*PLG*	-0.403	4.70E-23	Nature. 2018;558(7708):73–79	Blood plasma
			-0.438	2.10E-88	Nat Med. 2021;27(3):471-479.	Blood plasma
8q12.1	rs4737427	*PENK*	0.217	6.30E-10	Nature. 2018;558(7708):73–79	Blood plasma
11q12.1	rs11229011	*SERPING1*	-0.195	4.70E-08	Nature. 2018;558(7708):73–79	Blood plasma

© 2025. Martínez-Gutiérrez L et al. Published online at http://www.aginganddisease.org/EN/10.14336/AD.2025.0609

Supplementary Table 8. Enrichment results of prioritized genes in the Aging_Perturbations_from_GEO_up gene set library. Prioritized genes that are age-dysregulated in cell types relevant to GCA are shown.

Term	Overlap	P-value	OR	Combined Score	Genes
Human CD4+Tlyphocytes 52 years vs 72 years GSE62373 aging:182	6/270	0.002	4.96	31.01	*NAA60;ENOSF1;CALU;CISD2;TYMS;GTSF1*
Human CD4+Tlyphocytes 52 years vs 61 years GSE62373 aging:181	6/328	0.005	4.05	21.47	*NAA60;ENOSF1;THOC1;CALU;CISD2;GTSF1*
Human CD4+lyphocytes 52 years vs 72 years GSE62373 aging:167	5/232	0.005	4.76	25.02	*NAA60;MANBA;SETBP1;CHCHD7;GTSF1*
Human CD4+lyphocytes 29 years vs 40 years GSE62373 aging:152	4/279	0.045	3.10	9.59	*CD164;ALDH2;CISD2;NFKB1*
Human CD4+Tlyphocytes 61 years vs 72 years GSE62373 aging:169	3/231	0.100	2.78	6.41	*MANBA;SETBP1;COPZ1*
Mouse Granulocyte/Macrophage progenitors 6 weeks vs 88 weeks GSE48893 aging:223	3/248	0.117	2.59	5.56	*STARD6;TRIM59;PRG3*
Human CD4+lyphocytes 52 years vs 61 years GSE62373 aging:166	3/307	0.183	2.08	3.53	*MLKL;JADE2;GTSF1*
Human fibroblast 62 years vs 89 years GSE15829 aging:352	2/184	0.221	2.31	3.48	*PLG;GPN3*
Human CD4+Tlyphocytes 61 years vs 72 years GSE62373 aging:184	2/237	0.315	1.78	2.06	*TRAFD1;TIMM10*
Human dendriticcells(DCs) 20 years vs 77 years GSE58015 aging:187	2/290	0.407	1.45	1.30	*IRF5;NCKAP1L*
Human CD4+lyphocytes 29 years vs 52 years GSE62373 aging:153	2/317	0.451	1.32	1.05	*CD164;MXD4*
Human fibroblast 27 years vs 62 years GSE15829 aging:351	2/387	0.557	1.08	0.63	*SETBP1;BCL2L15*
Human CD4+lyphocytes 29 years vs 72 years GSE62373 aging:155	1/187	0.595	1.12	0.58	*SETBP1*
Human CD4+lyphocytes 52 years vs 81 years GSE62373 aging:168	1/210	0.638	0.99	0.45	*MANBA*
Human fibroblast 27 years vs 89 years GSE15829 aging:350	1/218	0.652	0.95	0.41	*BCL2L15*
Human CD4+Tlyphocytes 61 years vs 81 years GSE62373 aging:170	1/218	0.652	0.95	0.41	*ACTR2*
Human CD4+Tlymphocytes 25 years vs 64 years GSE62374 aging:129	1/221	0.657	0.94	0.40	*SPRED2*
Human CD4+lyphocytes 40 years vs 72 years GSE62373 aging:163	1/227	0.667	0.92	0.37	*SETBP1*
Human CD4+Tlyphocytes 52 years vs 81 years GSE62373 aging:183	1/235	0.679	0.88	0.34	*ENOSF1*
Human CD4+Tlyphocytes 40 years vs 52 years GSE62373 aging:177	1/241	0.689	0.86	0.32	*TIMM10*
Human CD4+Tlymphocytes 25 years vs 64 years GSE62374 aging:130	1/241	0.689	0.86	0.32	*HECTD4*
Human CD4+lyphocytes 40 years vs 61 years GSE62373 aging:160	1/242	0.690	0.86	0.32	*GTSF1*
Human CD4+Tlyphocytes 40 years vs 61 years GSE62373 aging:178	1/252	0.705	0.82	0.29	*GTSF1*
Human CD4+lyphocytes 29 years vs 61 years GSE62373 aging:154	1/258	0.713	0.80	0.27	*GTSF1*
Human CD4+lyphocytes 40 years vs 52 years GSE62373 aging:158	1/287	0.751	0.72	0.21	*TIMM10*
Human CD4+Tlyphocytes 29 years vs 40 years GSE62373 aging:172	1/291	0.756	0.71	0.20	*ALDH2*

© 2025. Martínez-Gutiérrez L et al. Published online at http://www.aginganddisease.org/EN/10.14336/AD.2025.0609

Supplementary Table 9. Enrichment results of prioritized genes in the Aging_Perturbations_from_GEO_down gene set library. Prioritized genes that are age-dysregulated in cell types relevant to GCA are shown.

Term	Overlap	P-value	Odds Ratio	Combined Score	Genes
Human CD4+Tlymphocytes 25 years vs 64 years GSE62374 aging:130	6/359	0.008	3.69	17.98	*MANBA;ALDH2;TIMM10;GPR84;TSPAN33;NFKB1*
Human CD4+lyphocytes 29 years vs 52 years GSE62373 aging:153	5/283	0.012	3.88	17.23	*NAA60;MANBA;ALDH2;GPR84;TYMS*
Human CD4+lyphocytes 29 years vs 72 years GSE62373 aging:155	6/413	0.015	3.19	13.50	*CBX5;ALDH2;TRIM59;GPR84;TYMS;TSPAN33*
Human CD4+lyphocytes 29 years vs 81 years GSE62373 aging:156	6/419	0.016	3.15	13.10	*NAA60;CBX5;PPTC7;TIMM10;GPR84;TSPAN33*
Human CD4+lyphocytes 40 years vs 52 years GSE62373 aging:158	5/313	0.017	3.50	14.14	*MANBA;ALDH2;SESN1;JADE2;GPR84*
Human CD4+lyphocytes 29 years vs 61 years GSE62373 aging:154	5/342	0.025	3.19	11.83	*ALDH2;PPTC7;HVCN1;TRIM59;TYMS*
Human CD4+Tlyphocytes 29 years vs 61 years GSE62373 aging:174	5/363	0.031	3.00	10.46	*FIG4;NFE2;ALDH2;NCKAP1L;TYMS*
Human CD4+Tlyphocytes 29 years vs 52 years GSE62373 aging:173	4/313	0.064	2.76	7.58	*MANBA;ALDH2;CALU;TYMS*
Human CD4+Tlyphocytes 40 years vs 52 years GSE62373 aging:177	4/359	0.094	2.39	5.65	*MANBA;ALDH2;ENOSF1;TYMS*
Human CD4+lyphocytes 40 years vs 72 years GSE62373 aging:163	4/373	0.105	2.30	5.19	*CBX5;ALDH2;GPR84;TSPAN33*
Human CD4+Tlymphocytes 25 years vs 64 years GSE62374 aging:129	4/379	0.109	2.26	5.01	*MANBA;SESN1;JADE2;TSPAN33*
Human CD4+Tlyphocytes 61 years vs 81 years GSE62373 aging:170	4/382	0.112	2.25	4.92	*NAA60;MLKL;TIMM10;GTSF1*
Human CD4+Tlyphocytes 40 years vs 61 years GSE62373 aging:178	3/348	0.234	1.83	2.66	*SMUG1;NFE2;ALDH2*
Human CD4+lyphocytes 40 years vs 61 years GSE62373 aging:160	3/358	0.247	1.78	2.49	*MANBA;ALDH2;GPR84*
Human CD4+Tlyphocytes 29 years vs 72 years GSE62373 aging:175	3/370	0.262	1.72	2.30	*NFE2;CBX5;ALDH2*
Human CD4+Tlyphocytes 72 years vs 81 years GSE62373 aging:171	3/373	0.266	1.70	2.25	*NAA60;SETBP1;TIMM10*
Human CD4+Tlyphocytes 29 years vs 81 years GSE62373 aging:176	3/377	0.271	1.68	2.20	*CBX5;PPTC7;TIMM10*
Human fibroblast 27 years vs 62 years GSE15829 aging:351	2/213	0.273	1.99	2.58	*PLG;GPN3*
Human CD4+lyphocytes 40 years vs 81 years GSE62373 aging:165	3/380	0.275	1.67	2.16	*ALDH2;GPR84;TSPAN33*
Human CD4+lyphocytes 52 years vs 81 years GSE62373 aging:168	3/390	0.288	1.63	2.02	*CD164;PPTC7;TIMM10*
Human fibroblast 62 years vs 89 years GSE15829 aging:352	3/416	0.322	1.52	1.72	*VPS29;PDE1B;KPNA4*
Human CD4+Tlymphocytes 25 years vs 64 years GSE62374 aging:131	2/294	0.413	1.43	1.26	*NFE2;ALDH2*
Human CD4+Tlyphocytes 29 years vs 40 years GSE62373 aging:172	2/309	0.438	1.36	1.12	*NCKAP1L;ACAD10*
Human dendriticcells(DCs) 20 years vs 77 years GSE58015 aging:187	2/310	0.440	1.35	1.11	*PRG2;TYMS*
Human CD4+Tlymphocytes 25 years vs 64 years GSE62374 aging:132	2/319	0.454	1.31	1.04	*NFE2;ALDH2*
Human CD4+Tlyphocytes 61 years vs 72 years GSE62373 aging:184	2/363	0.522	1.15	0.75	*ALDH2;SH2B3*
Human CD4+Tlyphocytes 40 years vs 81 years GSE62373 aging:180	2/365	0.525	1.15	0.74	*TRAP1;ALDH2*
Human CD4+Tlyphocytes 52 years vs 81 years GSE62373 aging:183	2/365	0.525	1.15	0.74	*CBX5;TIMM10*
Human fibroblast 27 years vs 89 years GSE15829 aging:350	2/382	0.550	1.09	0.65	*PDE1B;KPNA4*
Human CD4+Tlyphocytes 40 years vs 72 years GSE62373 aging:179	2/407	0.585	1.02	0.55	*ALDH2;SH2B3*
Human CD4+Tlyphocytes 52 years vs 61 years GSE62373 aging:181	1/272	0.732	0.76	0.24	*TIMM10*
Human CD4+lyphocytes 52 years vs 61 years GSE62373 aging:166	1/293	0.758	0.71	0.20	*PPTC7*
Human CD4+lyphocytes 29 years vs 40 years GSE62373 aging:152	1/321	0.789	0.64	0.15	*TIMM10*
Human CD4+Tlyphocytes 72 years vs 81 years GSE62373 aging:186	1/328	0.796	0.63	0.14	*TIMM10*
Human CD4+Tlyphocytes 52 years vs 72 years GSE62373 aging:182	1/330	0.798	0.63	0.14	*CBX5*
Mouse Granulocyte/Macrophage progenitors 6 weeks vs 88 weeks GSE48893 aging:223	1/352	0.819	0.59	0.12	*SMPD2*
Human CD4+lyphocytes 52 years vs 72 years GSE62373 aging:167	1/368	0.833	0.56	0.10	*TIMM10*
Human CD4+Tlyphocytes 61 years vs 72 years GSE62373 aging:169	1/369	0.833	0.56	0.10	*TRAFD1*

Supplementary Table 10. Enrichment results of prioritized genes in the GTEx Aging Signatures 2021 library. Prioritized genes that are age-dysregulated in cell types relevant to GCA are shown.

Term	Overlap	P-value	Odds Ratio	Combined Score	Genes
GTEx Blood 20-29 vs 60-69 Up	3/250	0.119	2.57	5.46	*PRG2;PLG;PRG3*
GTEx Blood 20-29 vs 30-39 Up	2/250	0.338	1.69	1.83	*SDR16C5;NFE2*
GTEx Blood 20-29 vs 40-49 Down	1/250	0.702	0.83	0.29	*NFE2*
GTEx Blood 20-29 vs 30-39 Down	1/250	0.702	0.83	0.29	*GTSF1*
GTEx BloodVessel 20-29 vs 70-79 Up	1/250	0.702	0.83	0.29	*PENK*
GTEx BloodVessel 20-29 vs 60-69 Up	1/250	0.702	0.83	0.29	*PENK*
GTEx Blood 20-29 vs 70-79 Up	1/250	0.702	0.83	0.29	*PLG*
GTEx BloodVessel 20-29 vs 40-49 Up	1/250	0.702	0.83	0.29	*PENK*
GTEx BloodVessel 20-29 vs 50-59 Up	1/250	0.702	0.83	0.29	*PENK*
GTEx Blood 20-29 vs 50-59 Up	1/250	0.702	0.83	0.29	*PLG*

© 2025. Martínez-Gutiérrez L et al. Published online at http://www.aginganddisease.org/EN/10.14336/AD.2025.0609

Supplementary Table 11. Results of the drug repurposing analysis.

Name	Status	Targets	Gene
N-[2-(2-methyl-1H-indol-3-yl)ethyl]thiophene-2-carboxamide	experimental	'ARPC1B', 'ACTR2', 'ACTR3', 'ARPC2', 'ARPC3', 'ARPC4', 'ARPC5'	*ACTR2*
(2S)-2-(3-bromophenyl)-3-(5-chloro-2-hydroxyphenyl)-1,3-thiazolidin-4-one	experimental	'ARPC1B', 'ACTR2', 'ACTR3', 'ARPC2', 'ARPC3', 'ARPC4', 'ARPC5'	*ACTR2*
Sulfasalazine	approved	'ALOX5', 'PTGS2', 'PTGS1', 'ACAT1', 'PLA2G1B', 'SLC7A11', 'NFKB1', 'NFKB2', 'IKBKB', 'CHUK', 'PPARG'	*NFKB1*
Pseudoephedrine	approved	'SLC6A2', 'SLC6A3', 'ADRA1A', 'ADRA2A', 'SLC6A4', 'ADRB2', 'ADRB1', 'NFATC1', 'TNF', 'NFKB1', 'ATF1', 'IL2'	*NFKB1*
Pranlukast	investigational	'CYSLTR1', 'TNF', 'IL5', 'RNASE3', 'NFKB1', 'MUC2', 'NFKB2'	*NFKB1*
HE3286	investigational	'NFKB1', 'NFKB2'	*NFKB1*
P54	investigational	'NFKB2', 'NFKB1'	*NFKB1*
NOX-700	investigational	'NFKB2', 'NFKB1'	*NFKB1*
SGN-30	investigational	'NFKB1', 'NFKB2'	*NFKB1*
Custirsen	investigational	'ESR1', 'NFKB2', 'NFKB1'	*NFKB1*
Andrographolide	investigational	'TNF', 'IL1B', 'IL6', 'NFKB2', 'NFKB1'	*NFKB1*
Triflusal	approved, investigational	'PTGS1', 'NFKB1', 'NOS2', 'PDE10A'	*NFKB1*
Guanosine-5'-Diphosphate	experimental	'HRAS', 'RHOA', 'TGM2', 'RAB11A', 'purA', 'ftsZ', 'TGM3', 'RAB8B', 'ARF6', 'ADSSL1', 'fusA', 'TUFM', 'der', 'gmm', 'engB', 'EIF2S3', 'GNAI1', 'ffh', 'GNAT1', 'tufB', 'RAB9A', 'NME1', 'RHEB', 'CDC42', 'tufA', 'ARF1', 'EEF2', 'relA', 'RAP2A', 'EFTUD1', 'ARL1', 'RAN', 'RAB7A', 'IRGC', 'GSPT1', 'RAB5A', 'EEF1A1', 'ARF4', 'GMDS', 'RALA', 'tuf', 'ARL5B', 'rab6', 'ARL3', 'NME2', 'gmd', 'RAC1', 'rsgA', 'SAR1B', 'Adss', 'ARL5A'	*SAR1B*
Alteplase	approved, investigational	'PLG', 'FGA', 'FGG', 'SERPINE1'	*PLG*
Urokinase	approved, investigational, withdrawn	'PLG', 'PLAUR', 'SERPINE1', 'SERPINB2', 'SERPINA5', 'LRP2', 'ST14', 'NID1'	*PLG*
Reteplase	approved, investigational	'PLG', 'FGA', 'SERPINE1'	*PLG*
Anistreplase	approved	'PLG', 'FGA', 'SERPINE1'	*PLG*
Tenecteplase	approved	'PLG', 'FGA', 'PLAUR', 'SERPINE1', 'SERPINB2', 'CLEC3B', 'KRT8', 'ANXA2', 'CALR', 'CANX', 'LRP1'	*PLG*
Streptokinase	approved, investigational	'PLG', 'F2R'	*PLG*
Tranexamic acid	approved	'PLG'	*PLG*
Aminocaproic acid	approved, investigational	'PLG', 'PLAT', 'LPA'	*PLG*
Bicine	experimental	'PLG', 'mltB', 'glf'	*PLG*
Desmoteplase	investigational	'PLG'	*PLG*
Aprotinin	approved, investigational, withdrawn	'PRSS1', 'CTRB1', 'PLG', 'KLK1'	*PLG*
Copper	approved, investigational	'APP', 'AHCY', 'HIST1H2BC', 'GAPDH', 'NME1', 'HIST1H1E', 'PRDX1', 'S100A8', 'RPSA', 'ACTG1', 'ENO1', 'EEF1A1', 'KRT8', 'P4HB', 'PDIA3', 'HSPD1', 'HSPA13', 'HSPA5', 'HSP90B1', 'TF', 'Lyar', 'RPS2', 'SRSF1', 'HNRNPA2B1', 'HNRNPH1', 'HNRNPH3', 'cbiD', 'HNRNPL', 'SFPQ', 'SF3A2', 'RACK1', 'ACTN1', 'ACY1', 'ANXA4', 'ANXA5', 'CALR', 'PKM', 'AKR1A1', 'CYB5R3', 'GSR', 'TKT', 'PRDX2', 'PRDX6', 'PPIA', 'HSPA8', 'HSP90AA1', 'PTGES3', 'STIP1', 'EIF6', 'EIF4A1', 'GPI', 'LDHA', 'PGK1', 'TUBA3C', 'TUBB', 'CFL1', 'YWHAB', 'GOT1', 'GSS', 'HDGF', 'IDH3A', 'CLIC1', 'PSME1', 'PEBP1', 'PGAM1', 'RANBP1', 'UGDH', 'B2M', 'SCO1', 'PRNP', 'GLRA1', 'HTT', 'NEIL1', 'NEIL2', 'HEPHL1', 'SERPINE1', 'S100A2', 'S100A4', 'SNCA', 'BDNF', 'PARK7', 'IAPP', 'TAC3', 'A1BG', 'AFM', 'AGT', 'AHSG', 'APCS', 'APOA1', 'APOA2', 'APOA4', 'APOBR', 'APOC2', 'APOC3', 'APOD', 'APOE', 'APOH', 'AZGP1', 'C1QC', 'C1S', 'C3', 'C4B', 'C4BPA', 'C5', 'C8B', 'C9', 'CFH', 'CFI', 'CLEC3B', 'CLU', 'F2', 'C1QBP', 'GSN', 'HBA1', 'HBB', 'CBX5', 'HPR', 'IGFALS', 'IGHG1', 'IGHG4', 'IGLL1', 'ITIH2', 'KNG1', 'KRT1', 'KRT10', 'KRT2', 'KRT9', 'LRG1', 'LUM', 'PGLYRP2', 'PLG', 'PON1', 'PPBP', 'SERPINA1', 'SERPINA4', 'SERPINA6', 'SERPINA7', 'SERPINC1', 'SERPIND1', 'SERPINF1', 'SERPINF2', 'SERPING1', 'TTR', 'VTN', 'APLP1'	*PLG*
Gabexate	investigational	'KLKB1', 'F2', 'PLG'	*PLG*
Aminocaproic acid	approved, investigational	'PLG', 'PLAT', 'LPA'	*LPA*
PPL-100	investigational	'SERPING1', 'SERPINA5', 'SERPINA1'	*SERPING1*
Copper	approved, investigational	'APP', 'AHCY', 'HIST1H2BC', 'GAPDH', 'NME1', 'HIST1H1E', 'PRDX1', 'S100A8', 'RPSA', 'ACTG1', 'ENO1', 'EEF1A1', 'KRT8', 'P4HB', 'PDIA3', 'HSPD1', 'HSPA13', 'HSPA5', 'HSP90B1', 'TF', 'Lyar', 'RPS2', 'SRSF1', 'HNRNPA2B1', 'HNRNPH1', 'HNRNPH3', 'cbiD', 'HNRNPL', 'SFPQ', 'SF3A2', 'RACK1', 'ACTN1', 'ACY1', 'ANXA4', 'ANXA5', 'CALR', 'PKM', 'AKR1A1', 'CYB5R3', 'GSR', 'TKT', 'PRDX2', 'PRDX6', 'PPIA', 'HSPA8', 'HSP90AA1', 'PTGES3', 'STIP1', 'EIF6', 'EIF4A1', 'GPI', 'LDHA', 'PGK1', 'TUBA3C', 'TUBB', 'CFL1', 'YWHAB', 'GOT1', 'GSS', 'HDGF', 'IDH3A', 'CLIC1', 'PSME1', 'PEBP1', 'PGAM1', 'RANBP1', 'UGDH', 'B2M', 'SCO1', 'PRNP', 'GLRA1', 'HTT', 'NEIL1', 'NEIL2', 'HEPHL1', 'SERPINE1', 'S100A2', 'S100A4', 'SNCA', 'BDNF', 'PARK7', 'IAPP', 'TAC3', 'A1BG', 'AFM', 'AGT', 'AHSG', 'APCS', 'APOA1', 'APOA2', 'APOA4', 'APOBR', 'APOC2', 'APOC3', 'APOD', 'APOE', 'APOH', 'AZGP1', 'C1QC', 'C1S', 'C3', 'C4B', 'C4BPA', 'C5', 'C8B', 'C9', 'CFH', 'CFI', 'CLEC3B', 'CLU', 'F2', 'C1QBP', 'GSN', 'HBA1', 'HBB', 'CBX5', 'HPR', 'IGFALS', 'IGHG1', 'IGHG4', 'IGLL1', 'ITIH2', 'KNG1', 'KRT1', 'KRT10', 'KRT2', 'KRT9', 'LRG1', 'LUM', 'PGLYRP2', 'PLG', 'PON1',	*SERPING1*

© 2025. Martínez-Gutiérrez L et al. Published online at http://www.aginganddisease.org/EN/10.14336/AD.2025.0609

		'PPBP', 'SERPINA1', 'SERPINA4', 'SERPINA6', 'SERPINA7', 'SERPINC1', 'SERPIND1', 'SERPINF1', 'SERPINF2', 'SERPING1', 'TTR', 'VTN', 'APLP1'	
Gefapixant	approved, investigational	'P2RX3', 'P2RX2'	*P2RX3*
Sargramostim	approved, investigational	'CSF2RA', 'IL3RA', 'CSF2RB', 'SDC2', 'PRG2'	*PRG2*
Chymopapain	approved, withdrawn	'PRG2'	*PRG2*
Copper	approved, investigational	'APP', 'AHCY', 'HIST1H2BC', 'GAPDH', 'NME1', 'HIST1H1E', 'PRDX1', 'S100A8', 'RPSA', 'ACTG1', 'ENO1', 'EEF1A1', 'KRT8', 'P4HB', 'PDIA3', 'HSPD1', 'HSPA13', 'HSPA5', 'HSP90B1', 'TF', 'Lyar', 'RPS2', 'SRSF1', 'HNRNPA2B1', 'HNRNPH1', 'HNRNPH3', 'cbiD', 'HNRNPL', 'SFPQ', 'SF3A2', 'RACK1', 'ACTN1', 'ACY1', 'ANXA4', 'ANXA5', 'CALR', 'PKM', 'AKR1A1', 'CYB5R3', 'GSR', 'TKT', 'PRDX2', 'PRDX6', 'PPIA', 'HSPA8', 'HSP90AA1', 'PTGES3', 'STIP1', 'EIF6', 'EIF4A1', 'GPI', 'LDHA', 'PGK1', 'TUBA3C', 'TUBB', 'CFL1', 'YWHAB', 'GOT1', 'GSS', 'HDGF', 'IDH3A', 'CLIC1', 'PSME1', 'PEBP1', 'PGAM1', 'RANBP1', 'UGDH', 'B2M', 'SCO1', 'PRNP', 'GLRA1', 'HTT', 'NEIL1', 'NEIL2', 'HEPHL1', 'SERPINE1', 'S100A2', 'S100A4', 'SNCA', 'BDNF', 'PARK7', 'IAPP', 'TAC3', 'A1BG', 'AFM', 'AGT', 'AHSG', 'APCS', 'APOA1', 'APOA2', 'APOA4', 'APOBR', 'APOC2', 'APOC3', 'APOD', 'APOE', 'APOH', 'AZGP1', 'C1QC', 'C1S', 'C3', 'C4B', 'C4BPA', 'C5', 'C8B', 'C9', 'CFH', 'CFI', 'CLEC3B', 'CLU', 'F2', 'C1QBP', 'GSN', 'HBA1', 'HBB', 'CBX5', 'HPR', 'IGFALS', 'IGHG1', 'IGHG4', 'IGLL1', 'ITIH2', 'KNG1', 'KRT1', 'KRT10', 'KRT2', 'KRT9', 'LRG1', 'LUM', 'PGLYRP2', 'PLG', 'PON1', 'PPBP', 'SERPINA1', 'SERPINA4', 'SERPINA6', 'SERPINA7', 'SERPINC1', 'SERPIND1', 'SERPINF1', 'SERPINF2', 'SERPING1', 'TTR', 'VTN', 'APLP1'	*CBX5*
Caffeine	approved	'ADORA1', 'ADORA2A', 'ADORA2B', 'ADORA3', 'PDE1B', 'PDE4B', 'RYR1', 'PDE1A', 'PRKDC', 'PIK3CD', 'PIK3CA', 'PIK3CB', 'ITPR1', 'ATM'	*PDE1B*
Nicardipine	approved, investigational	'CACNA1C', 'CACNB2', 'CACNA2D1', 'CACNA1D', 'PDE1A', 'PDE1B', 'ADRA1A', 'ADRA1B', 'ADRA1D', 'CHRM1', 'CHRM2', 'CHRM3', 'CHRM4', 'CHRM5', 'CALM1', 'CACNG1', 'CACNA1G', 'CACNA1H', 'CACNA1I'	*PDE1B*
Felodipine	approved, investigational	'CACNA1C', 'CACNA2D1', 'CACNB2', 'CACNA1D', 'CACNA1S', 'CACNA1H', 'CACNA2D2', 'CALM1', 'PDE1B', 'PDE1A', 'NR3C2', 'TNNC2', 'TNNC1'	*PDE1B*
Papaverine	approved, investigational	'PDE4B', 'PDE10A', 'PDE1B'	*PDE1B*
Bepridil	approved, withdrawn	'CACNA1A', 'CACNA1H', 'CACNA2D2', 'ATP1A1', 'KCNQ1', 'TNNC1', 'CALM1', 'PDE1B', 'PDE1A', 'KCNH2'	*PDE1B*
S,S-(2-Hydroxyethyl)Thiocysteine	experimental	'thyA', 'PDE4B', 'TYMS', 'PRKACA', 'PIM1', 'ARG1', 'lacZ', 'icd', 'SERPINB5', 'malE', 'hemB', 'rmlC', 'PDE1B', 'SNX3', 'licT', 'CYTH2'	*PDE1B*
Pazopanib	approved	'FLT1', 'KDR', 'FLT4', 'PDGFRA', 'PDGFRB', 'KIT', 'FGFR3', 'ITK', 'FGF1', 'SH2B3'	*SH2B3*
NADH	approved, nutraceutical	'UGDH', 'ADH1A', 'SORD', 'ADH4', 'ADH1C', 'ADH1B', 'ADH7', 'GPD1', 'LDHAL6A', 'ADH5', 'AKR1B1', 'HSD17B10', 'MDH2', 'LDHA', 'ME2', 'HSD17B4', 'LDHAL6B', 'LDHC', 'LDHB', 'EHHADH', 'HADH', 'ME3', 'BDH1', 'MDH1', 'HIBADH', 'HSD17B3', 'HMGCR', 'ME1', 'PHGDH', 'HSD17B7', 'IDH3G', 'IDH3B', 'IDH3A', 'HSD17B1', 'HSD17B2', 'RDH5', 'H6PD', 'HSD17B8', 'AKR1C4', 'IMPDH2', 'AKR1C3', 'HPGD', 'TSTA3', 'AKR1C1', 'HSD11B1', 'HSD3B2', 'HSD3B1', 'IMPDH1', 'HADHA', 'ALDH2', 'HSD11B2', 'AKR1C2', 'NSDHL', 'ALDH3B2', 'ALDH6A1', 'ALDH3A1', 'ALDH1A1', 'GAPDHS', 'ALDH9A1', 'ALDH1A3', 'ALDH1B1', 'ALDH3B1', 'GAPDH', 'ALDH1A2', 'ALDH3A2', 'ALDH5A1', 'ALDH7A1', 'BLVRB', 'PYCR2', 'PYCR1', 'GLUD1', 'PDHB', 'PDHA2', 'PDHA1', 'BLVRA', 'ACADS', 'DHFR', 'OGDH', 'GLUD2', 'DHCR7', 'NQO2', 'MT-ND2', 'AASS', 'MT-ND3', 'NDUFS7', 'MTHFD1', 'MTHFD2', 'ALDH4A1', 'CYB5R3', 'NDUFA11', 'MT-ND4', 'QDPR', 'MT-ND1', 'NNT', 'NDUFA5', 'NDUFA9', 'MT-ND4L', 'NDUFA10', 'NDUFA7', 'NDUFA1', 'MT-ND6', 'MT-ND5', 'NDUFA4', 'NDUFA6', 'NDUFAB1', 'NDUFA2', 'NDUFA8', 'NDUFA3', 'NDUFB8', 'NDUFC2', 'NDUFB1', 'NDUFS1', 'NDUFB10', 'NDUFB4', 'NDUFB3', 'NDUFB2', 'NDUFB7', 'NDUFB9', 'NDUFS2', 'NDUFB5', 'NDUFC1', 'NDUFB6', 'NDUFV3', 'DLD', 'NDUFS3', 'GSR', 'NDUFA12', 'NDUFS5', 'NDUFS4', 'NDUFV1', 'NDUFV2', 'NDUFA13', 'CDO1', 'NDUFS6', 'NDUFA4L2', 'NDUFS8', 'CYP4A11', 'DLAT', 'AMT', 'MSMO1', 'HMOX1', 'HMOX2', 'CYP17A1', 'TYR'	*ALDH2*
Guanidine	approved	'ALDH2', 'RNASE1', 'DLG4', 'E', 'GAMT', 'rocF'	*ALDH2*
Disulfiram	approved	'DBH', 'ALDH2'	*ALDH2*
Daidzin	experimental	'ALDH2'	*ALDH2*
Crotonaldehyde	experimental	'ALDH2'	*ALDH2*
Grn163l	investigational	SMG6', 'SCYL1', 'PTGES3', 'TERT'	*SMG6*
Pyridoxal phosphate	approved, investigational, nutraceutical	'AGXT2', 'GAD1', 'CBS', 'KYNU', 'SHMT1', 'NFS1', 'GOT1', 'OAT', 'ODC1', 'AADAT', 'ABAT', 'PNPO', 'SGPL1', 'TAT', 'CCBL1', 'SPTLC2', 'CSAD', 'HDC', 'AZIN2', 'SDS', 'GCAT', 'GLDC', 'GPT', 'PSAT1', 'ALAS1', 'AGXT', 'PDXP', 'SPTLC1', 'CTH', 'BCAT1', 'BCAT2', 'PROSC', 'GOT2', 'PYGB', 'PYGM', 'DDC', 'GAD2', 'CCBL2', 'GADL1', 'SCLY', 'IGSF10', 'PHYKPL', 'SHMT2', 'ALAS2', 'GPT2', 'MOCOS', 'SDSL', 'SRR', 'SEPSECS', 'SPTLC3', 'GAD65', 'GOT1'	*SRR*
Serine	investigational, nutraceutical	'CBS', 'SDS', 'SRR', 'SPTLC2', 'SPTLC1', 'SARS', 'AGXT'	*SRR*
Raltitrexed	approved, investigational	'TYMS', 'FPGS'	*TYMS*
Floxuridine	approved	'TYMS'	*TYMS*
Trifluridine	approved, investigational	'TYMS'	*TYMS*
Gemcitabine	approved	'RRM1', 'TYMS', 'CMPK1'	*TYMS*
Fluorouracil	approved	'TYMS'	*TYMS*
Methotrexate	approved	'TYMS', 'ATIC', 'DHFR'	*TYMS*
Pemetrexed	approved, investigational	'TYMS', 'ATIC', 'DHFR', 'GART'	*TYMS*
Capecitabine	approved, investigational	'TYMS'	*TYMS*

© 2025. Martínez-Gutiérrez L et al. Published online at http://www.aginganddisease.org/EN/10.14336/AD.2025.0609

Thymidine monophosphate	experimental	'tmk', 'DTYMK', 'TYMS', 'topA', 'thyA', 'dnaQ', 'rmlA'	*TYMS*
10-Propargyl-5,8-Dideazafolic Acid	experimental	'NQO2', 'TYMS', 'DHRS4L1', 'thyA', 'thyA', 'THYA'	*TYMS*
Deoxyuridine monophosphate	experimental	'dut', 'thyA', 'thyA', 'TYMS', 'thyX', 'pol', 'dut', 'THYA'	*TYMS*
S,S-(2-Hydroxyethyl)Thiocysteine	experimental	'thyA', 'PDE4B', 'TYMS', 'PRKACA', 'PIM1', 'ARG1', 'lacZ', 'icd', 'SERPINB5', 'malE', 'hemB', 'rmlC', 'PDE1B', 'SNX3', 'licT', 'CYTH2'	*TYMS*
Thymectacin	investigational	'TYMS'	*TYMS*
ANX-510	investigational	'TYMS'	*TYMS*
OSI-7904L	investigational	'TYMS'	*TYMS*
Pralatrexate	approved, investigational	'DHFR', 'TYMS', 'FPGS'	*TYMS*
2,4-Diamino-5-phenyl-6-ethylpyrimidine	experimental	'TYMS'	*TYMS*
N-[2-Chloro-5-(trifluoromethyl)phenyl]imidodicarbonimidic diamide	experimental	'TYMS'	*TYMS*
N-(3,5-dimethoxyphenyl)imidodicarbonimidic diamide	experimental	'TYMS'	*TYMS*
6,6-DIMETHYL-1-[3-(2,4,5-TRICHLOROPHENOXY)PROPOXY]-1,6-DIHYDRO-1,3,5-TRIAZINE-2,4-DIAMINE	experimental	'TYMS'	*TYMS*
Tegafur	approved, investigational	'TYMS'	*TYMS*
Tegafur-uracil	approved, investigational	'DPYD', 'TYMS'	*TYMS*

© 2025. Martínez-Gutiérrez L et al. Published online at http://www.aginganddisease.org/EN/10.14336/AD.2025.0609

SUPPLEMENTARY DATA

Appendix

INTERNATIONAL GCA CONSORTIUM

Spain
Santos Castañeda, Roser Solans, Jaume Mestre-Torres, Norberto Ortego-Centeno, José Luis Callejas, Luis Caminal-Montero, Marc Corbera-Bellalta, Eugenio de Miguel, J. Bernardino Díaz- López, María Jesús García-Villanueva, Carmen Gómez-Vaquero, Mercedes Guijarro-Rojas, Ana Hidalgo-Conde, Begoña Marí-Alfonso, Agustín Martínez-Berriochoa, Inmaculada C. Morado, Javier Narváez, Marc Ramentol-Sintas, Aleida Martínez-Zapico, Víctor Manuel Martínez- Taboada, José A. Miranda-Filloy, Jordi Monfort, Mercedes Pérez-Conesa, Sergio Prieto- González, Enrique Raya, Raquel Ríos-Fenández, Julio Sánchez-Martín, Bernardo Sopeña, Laura Tío, Ainhoa Unzurrunzaga.

North America and Canada (Vasculitis Clinical Research Consortium)
Peter A Merkel, Nader Khalidi, Carol A. Langford, Steven Ytterberg, Simon Carette, Sharon Chung, David Cuthbertson, Lindsy J. Forbess, Ora Gewurz-Singer, Gary S. Hoffman, Curry L. Koening, Kathleen M. Maksimowicz-McKinnon, Carol A. McAlear, Larry W. Moreland, Christian Pagnoux, Philip Seo, Ulrich Specks, Robert F. Spiera, Antoine Sreih, Kenneth J. Warrington, Paul A. Monach, Michael Weisman.

Italy
Augusto Vaglio, Lorenzo Beretta, Marcello Govoni, Giacomo Emmi, Marco A Cimmino, Carlo Salvarani.

Germany
Torsten Witte, Thomas Neumann, Julia Holle, Verena Schönau, Peter Lamprecht, Sebastian Klapa.

France
Gregory Pugnet, Thomas Papo, Julien Haroche, Alfred Mahr, Luc Mouthon.

Norway
Øyvind Molberg, Andreas P. Diamantopoulo.

Netherlands
Alexandre Voskuyl, Yannick van Sleen, Elisabeth Brouwer.

Switzerland
Thomas Daikeler, Christoph T. Berger.

Ireland
Eamonn S. Molloy.

© 2025. Martínez-Gutiérrez L et al. Published online at http://www.aginganddisease.org/EN/10.14336/AD.2025.0609

UK GCA CONSORTIUM

Sarah L Mackie, Louise Sorensen, Lubna Haroon Raashid, Steve Martin, James I Robinson, Oliver Wordsworth, Isobel Whitwell, Jessica Brock, Victoria Douglas, Chamila Hettiarachchi, Jacqui Bartholomew, Stephen Jarrett, Gayle Smithson, Michael Green, Pearl Clark Brown, Cathy Lawson, Esther Gordon, Suzanne Lane, Rebecca Francis, Bhaskar Dasgupta, Bridgett Masunda, Jo Calver, Yusuf Patel, Charlotte Thompson, Louise Gregory, Sarah Levy, Ajit Menon, Amy Thompson, Lisa Dyche, Michael Martin, Charles Li, Ramasharan Laxminarayan, Louise Wilcox, Ralph de Guzman, John Isaacs, Alice Lorenzi, Gary Reynolds, Ross Farley, Helain Hinchcliffe- Hume, Victoria Bejarano, Susan Hope, Pradip Nandi, Lynne Stockham, Catherine Wilde, Donna Durrant, Mark Lloyd, Chee-Seng Ye, Rob Stevens, Amjad Jilani, David Collins, Suzannah Pegler, Ali Rivett, Liz Price, Neil McHugh, Sarah Skeoch, Diana O'Kane, Sue Kirkwood, Saravanan Vadivelu, Susan Pugmire, Shabina Sultan, Emma Dooks, Lisa Armstrong, Hala Sadik, Anupama Nandagudi, Tolu Abioye, Angelo Ramos, Steph Gumus, Nidhi Sofat, Abiola Harrison, Abi Seward, Susan Mollan, Ray Rahan, Helen Hawkins, Hedley Emsley, Anna Bhargava, Vicki Fleming, Marianne Hare, Sonia Raj, Emmanuel George, Nicola Allen, Karl Hunter, Eoin O'Sullivan, Georgina Bird, Malgorzata Magliano, Katarina Manzo, Bobbie Sanghera, David Hutchinson, Fiona Hammonds, Poonam Sharma, Richard Cooper, Graeme McLintock, Zaid S. Al-Saffar, Mike Green, Kerry Elliott, Tania Neale, Janine Mallinson, Peter Lanyon, Marie- Josephe Pradere, Natasha Jordan, Ei Phyu Htut, Thelma Mushapaidzi, Donna Abercrombie, Sam Wright, Jane Rowlands, Chetan Mukhtyar, James Kennedy, Damodar Makkuni, Elva Wilhelmsen, Michael Kouroupis, Shweta Bhagat, Lily John, Rod Hughes, Margaret Walsh, Marie Buckley, Kirsten Mackay, Tracey Camden-Woodley, Joan Redome, Kirsty Pearce, Thiraupathy Marianayagam, Carina Cruz, Elizabeth Warner, Ishmael Atchia, Claire Walker, Karen Black, Stacey Duffy, Marwan Bukhari, Lynda Fothergill, Rebecca Jefferey, Jackie Toomey, Ceril Rhys Dillon, Carla Pothecary, Lauren Green, Tracey Toms, Linda Maher, Diana Davis, Amrinder Sayan, Mini Thankachen, Mahdi Abusalameh, Jessica Record, Asad Khan, Sam Stafford, Azza Hussein, Clare Williams, Alison Fletcher, Laura Johson, Richard Burnett, Robert Moots, Helen Frankland, James Dale, Karen Black, Kirsten Moar, Carol Hollas, Ben Parker, Derek Ridings, Sandhya Eapen, Sindhu John, Jo Robson, Lucy Belle Guthrie, Rose Fyfe, Moira Tait, Jonathan Marks, Emma Gunter, Rochelle Hernandez, Smita Bhat, Paul Johnston, Muhammad Khurshid, Charlotte Barclay, Deepti Kapur, Helen Jeffrey, Anna Hughes, Lauren Slack, Eleri Thomas, Anna Royon, Angela Hall, Jon King, Sindi Nyathi, Vanessa Morris, Madhura Castelino, Ellie Hawkins, Linda Tomson, Animesh Singh, Annalyn Nunag, Stella O'Connor, Nathan Rushby, Nicola Hewitson, Kenny O'Sunmboye, Adam Lewszuk, Louise Boyles, Martin Perry, Emma Williams, Christine Graver, Emmanuel Defever, Sanjeet Kamanth, Dominic Kay, Joe Ogor, Louise Winter, Sarah Horton, Gillian Welch, Kath Hollinshead, James Peters, Julius Labao, Andrea Dmello, Julie Dawson, Denise Graham, Denise De Lord, Jo Deery, Tracy Hazelton.
